# Allosteric and Electrostatic Cooperativity in a Heteroditopic Halogen Bonding Receptor System

**DOI:** 10.1002/asia.202201170

**Published:** 2022-12-28

**Authors:** Andrew J. Taylor, Andrew Docker, Paul D. Beer

**Affiliations:** ^1^ Chemistry Research Laboratory Department of Chemistry University of Oxford 12 Mansfield Road Oxford OX1 3TA UK

**Keywords:** allosteric, cooperativity, halogen bonding, heteroditopic, ion-pair

## Abstract

A halogen bonding (XB) heteroditopic receptor, consisting of a 1,3‐bis‐iodo‐triazole benzene XB anion binding site covalently appended via a flexible methylene group with two benzo‐15‐crown‐5 (B15C5) cation binding moieties, and its hydrogen bonding receptor analogue, are used to delineate the mechanisms of cooperativity for alkali metal halide ion‐pair recognition. Extensive cation, anion and ion‐pair ^1^H NMR titration investigations demonstrate the combination of allosteric pre‐organisation, via 1 : 1 stoichiometric intramolecular potassium and rubidium metal cation bis‐B15C5 sandwich complexation, in concert with favourable electrostatics and XB potency, results in a remarkable enhancement of halide anion binding affinity by a factor of at least 700. By contrast, a notably diminished halide anion affinity enhancement factor of just 15 is observed with the corresponding 1 : 1 stoichiometric sodium cation complexed receptor system, where the smaller sized cation singly occupies one B15C5 unit and only an electrostatic contribution to cooperativity is possible. These observations serve to illustrate that allosteric pre‐organisation capability, electrostatic attraction and XB mediated anion recognition are important strategic design features to incorporate in future high‐fidelity heteroditopic ion‐pair receptor development.

## Graphical abstract

**The mechanisms of cooperativity** for alkali metal halide ion‐pair recognition by a novel halogen bonding (XB) heteroditopic receptor are delineated via extensive cation, anion and ion‐pair ^1^H NMR titration investigations. Through the contrasting size‐dictated alkali metal cation binding modes of the XB receptor, these results serve to highlight the importance of combining allosteric preorganisation, electrostatics and XB potency to maximize enhancement of halide anion binding affinity of the pre‐metal cation complexed XB receptor.

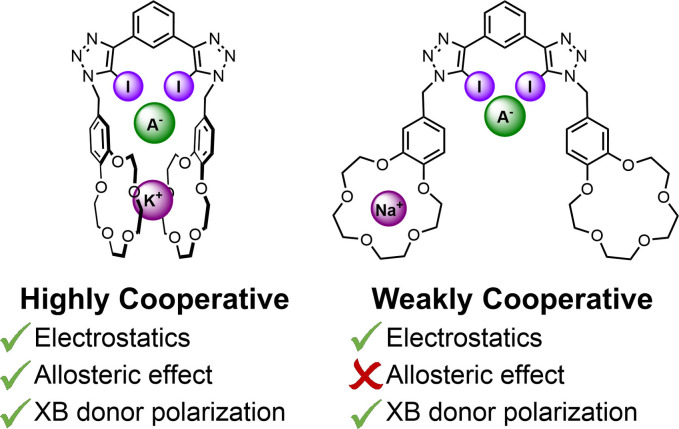

## Introduction

In the field of ion recognition, the design and construction of heteroditopic receptors, capable of simultaneously binding cations and anions, or ion‐pairs, continue to garner much attention.[^1^, ^2^] This is largely due to their frequently enhanced binding affinities^[3]^ and modulated selectivity profiles,^[4]^ relative to their monotopic analogues. Indeed, instances where an ion binding event enhances the association of its counterion, referred to as positive cooperativity,[^5^, ^6^] have realized notable achievements in the development of highly potent and selective heteroditopic molecular recognition systems, facilitating use in salt extraction^[7]^ or solubilization,[^8^, ^9^, ^10^] membrane transport and zwitter‐ion binding applications.[^11^, ^12^]

Typically two dominant mechanisms are used to rationalise positive cooperative behaviour exhibited by heteroditopic receptors: electrostatic[^7^, ^13^, ^14^] and allosteric effects.[^10^, ^15^, ^16^] In the former, enhancement is ascribed to the inherent Coulombic attraction between the proximal co‐bound, oppositely‐charged ions, whilst in the latter, an ion binding event induces a conformational change in the receptor which promotes counterion association. Whilst a fundamental and detailed understanding of these effects is critical to their considered incorporation into ion‐pair host design, reliable delineation of their relative contributions has proven challenging due to the inherent complexities in tracking multiple ionic species across complex equilibria pathways.^[17]^

We and others have demonstrated that anion recognition strategies employing exotic sigma‐hole based interactions,[^18^, ^19^, ^20^, ^21^, ^22^, ^23^] including halogen bonding (XB),[^24^, ^25^, ^26^, ^27^, ^28^, ^29^, ^30^, ^31^] engender considerable advantages over more traditional approaches, such as hydrogen bonding (HB). Furthermore, while rare, the few reports[^32^, ^33^, ^34^, ^35^, ^36^, ^37^] of XB integration in heteroditopic receptor design have also revealed unique behavior. In particular, XB mediated anion binding potency can be effectively ‘switched on’ by an inductive effect of a co‐bound alkali metal cation, polarizing a proximal C−I XB donor, which constitutes a novel and interesting mechanism of cooperativity.^[38]^ Motivated by these findings, we sought to exploit the combination of electrostatic, allosteric and XB donor polarization cooperativity mechanisms in the design of a novel, potentially potent heteroditopic receptor system.

**Figure 1:**
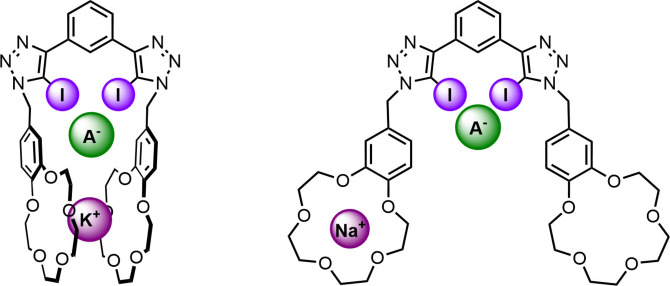
Contrasting binding modes of ion‐pair host **1 ⋅ XB**.

Herein, we report a heteroditopic receptor, **1 ⋅ XB**, capable of alkali metal halide ion‐pair recognition, comprising of a 1,3‐bis‐iodo‐triazole benzene XB anion binding site, appended via a methylene linker with two benzo‐15‐crown‐5 (B15C5) cation binding moieties (Figure 1). Extensive ^1^H NMR cation, anion and ion‐pair titration experiments not only demonstrate the starkly contrasting potassium and sodium cation complexation modes and stoichiometries of the XB receptor, but critically also show that these cation binding modes profoundly influence the relative contributions of electrostatic and allosteric ion‐pair recognition cooperativity. Importantly, these studies offer a rare systematic insight into relative mechanistic contributions to positive cooperativity and their interplay in heteroditopic ion‐pair receptors.

## Results and Discussion

### Receptor Design and Synthesis

**Figure 2:**
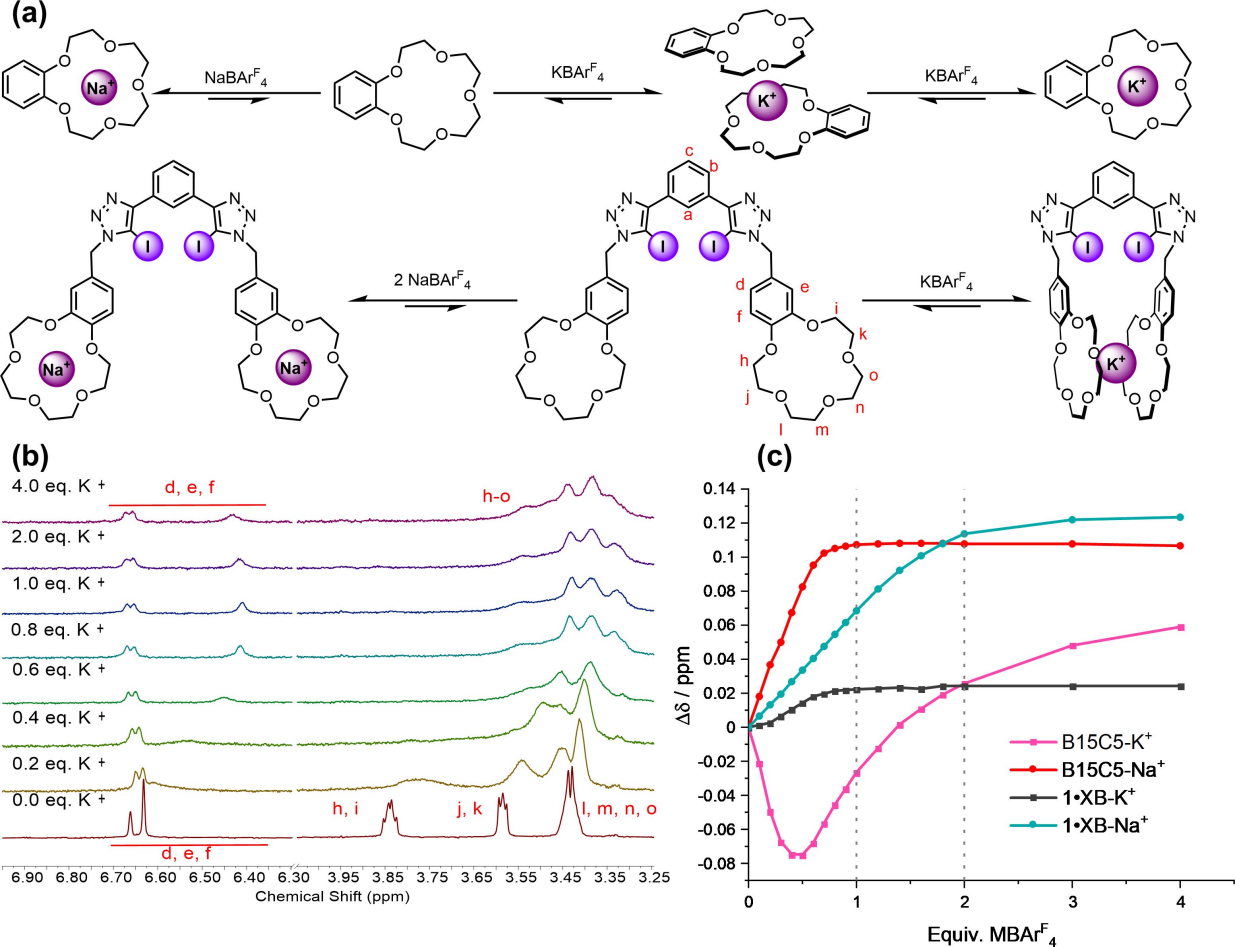
(a) Contrasting cation binding modes of **1 ⋅ XB** and B15C5 with Na^+^ and K^+^. (b) Stacked, truncated ^1^H NMR spectra of **1 ⋅ XB** upon addition of KBAr^F^_4_. (c) Plot of the change in chemical shift of the crown‐ether aromatic protons of **1 ⋅ XB** and B15C5 upon addition of KBAr^F^_4_ and NaBAr^F^_4_.

**Scheme 1:**
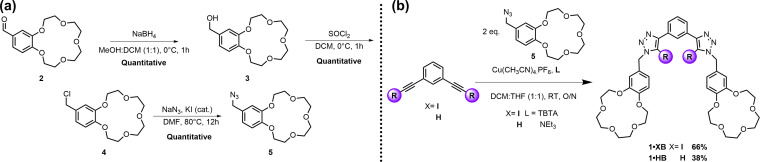
Synthesis of ion‐pair hosts **1 ⋅ XB** and **1 ⋅ HB**.

The target XB heteroditopic ion‐pair receptor design featured a potent bidentate 1,3‐bis‐iodo‐triazole benzene XB donor motif appended with methylene‐linked benzo‐15‐crown‐15 (B15C5) cation binding units. It was anticipated that the incorporation of the flexible methylene unit would allow for two possible cation binding modes, dependent on the size of the alkali metal cation (Figure 2a), either (i) a 1 : 1 stoichiometric sandwich‐type complex in which both B15C5 units engage with the cation in an intramolecular fashion or (ii) 1 : 2 host‐guest stoichiometric cation complexation, where each B15C5 unit binds a single metal cation. The target XB ion‐pair host was synthesised via a copper(I)‐catalysed azide‐alkyne cycloaddition (CuAAC) methodology (Scheme 1).

The requisite methylene‐azide appended B15C5 **5** was synthesized according to Scheme 1a. Reduction of 4’‐formylbenzo‐15‐crown‐5^[39]^ **2** in CH_2_Cl_2_:MeOH mixtures was accomplished with NaBH_4_ to afford the corresponding alcohol **3** in excellent yield. A chlorination reaction of **3**, by treatment with SOCl_2_, gave the alkyl chloride derivative **4**, which was subsequently subjected to an S_N_2 substitution reaction with NaN_3_ in DMF to afford the azide **5** in quantitative yield over three steps, without the need for column chromatography.

With azide **5** in hand, typical CuAAC reaction conditions were employed for the synthesis of **1 ⋅ XB** (Scheme 1b). Specifically, 2 equivalents of **5** were stirred with 1,3‐bis‐iodoethynylbenzene^[40]^ in the presence of catalytic [Cu(MeCN)_4_]PF_6_ and the rate accelerating ligand TBTA. After stirring at ambient temperature for 12 hours, TLC analysis of the reaction mixture showed complete consumption of the precursor alkyne and the reaction mixture was subjected to a NH_4_OH/EDTA aqueous work up procedure. Interestingly, attempts to purify the crude mixture via silica gel column chromatography proved consistently unsuccessful. Whilst initially surprising, this presumably reflects the instability of the triazole‐methylene‐aromatic linkage, particularly in the presence of the silica‐gel stationary phase. Consequently, purification by iterative trituration with mixtures of methanol and diethyl ether was used, which afforded the target host **1 ⋅ XB** in 66% yield.

To elucidate the role of XB interactions in the potential ion‐pair recognition behaviour of **1 ⋅ XB**, the corresponding HB receptor, **1 ⋅ HB**, was also synthesized via an analogous procedure, starting from 1,3‐bis‐ethynylbenzene, although replacement of TBTA with triethylamine was required to allow for purification of **1 ⋅ HB** by trituration, which was isolated in 38% yield.

Both ion‐pair hosts **1 ⋅ XB** and **1 ⋅ HB** were characterized by ^1^H and ^13^C NMR and high‐resolution electrospray ionization mass spectrometry (ESI‐MS).

### Cation Binding Studies

The sodium and potassium cation binding modes and stoichiometries of B15C5 were determined initially through 1H NMR titration experiments. In a typical experiment, aliquots of Na^+^ and K^+^ as their organic solvent‐soluble tetrakis 3,5‐bis(trifluoromethyl)phenyl borate (BAr_4_^F−^) salts were added to a 1 : 1 CD_3_CN/CDCl_3_ (v/v) solution of B15C5. In the case of the NaBAr^F^_4_ titration, the addition of increasing equivalents of Na^+^ induced notable downfield shift perturbations in the methylene and aryl proton signals, which sharply ceased beyond 1 equivalent of Na^+^, consistent with the formation of a highly stable (*K_a_* (Na^+^)>10^5^ M^−1^) 1 : 1 stoichiometric complex (Figure 2c).^[41]^ Contrastingly, addition of KBAr^F^_4_ elicited upfield perturbations in the same proton signals, concomitant with pronounced broadening, reaching a maximum upfield perturbation at 0.5 equivalents of K^+^. Further addition of K^+^ beyond 0.5 equivalents resulted in a reversal of the direction of chemical shift (Figure 2c). Consistent with the documented propensity of larger alkali metal cations to form higher stoichiometry complexes with B15C5,[^42^, ^43^, ^44^, ^45^] this more complex titration isotherm profile can be confidently attributed to the formation of 2 : 1 stoichiometric B15C5‐K^+^ intermolecular sandwich complex at sub‐equimolar K^+^ concentrations, which, upon further addition of K^+^, dissociates and a 1 : 1 B15C5‐K^+^ complex becomes the dominant species (Figure 2a). Furthermore, from visual inspection of the isotherm profile it is estimated that the *K_a_* values associated with the formation of the 2 : 1 and 1 : 1 stoichiometric complexes, *K*_*2:1*_ and *K*_*1:1*_ are also both greater than 10^5^ M^−1^ in the 1 : 1 CD_3_CN/CDCl_3_ (v/v) mixed solvent.

Analogous Na^+^ and K^+^ binding ^1^H NMR titration experiments were subsequently undertaken with **1 ⋅ XB**. As for B15C5, the addition of Na^+^ to a 1 : 1 CD_3_CN/CDCl_3_ (v/v) solution of **1 ⋅ XB** induced downfield perturbations of the crown‐ether derived methylene and aryl proton signals, which exhibited the largest‐magnitude chemical shift changes between 0 and 2 equivalents of Na^+^, suggesting one sodium cation binding to each B15C5 unit of the heteroditopic receptor. Although the Na^+^ binding strength was too high to be accurately determined by ^1^H NMR titration experiments, these observations are consistent with a 1 : 2 stoichiometric host‐guest complex wherein both Na^+^ *K_a_* values are in excess of 10^5^ M^−1^.

Upon addition of KBAr^F^_4_, dramatic changes in the proton signals of **1 ⋅ XB** were observed, most conspicuously significant broadening of the crown‐ether methylene signals (Figure 2b), which became increasingly resolved at equimolar K^+^ and exhibited no further change upon further potassium addition, in notable contrast to the B15C5‐KBAr^F^_4_ titration experiments. These observations, in concert with the strong shielding effects also seen, support the formation of an intramolecular 1 : 1 stoichiometric co‐facial K^+^ bis‐B15C5 sandwich complex.^[4]^ Whilst the very high K^+^ binding affinity precluded quantitative determination of a *K_a_* (K^+^) binding constant, it is noteworthy that the diagnostic ^1^H NMR spectroscopic evidence for the sandwich complex persisted in the presence of excess K^+^, unlike the behavior observed for B15C5, reflecting the additional stability conferred by the intramolecular nature of the **1 ⋅ XB**‐K^+^ complex.

### Anion Binding Studies

With evidence for the contrasting binding modes of Na^+^ and K^+^ established, attention turned to quantifying the anion binding properties of **1 ⋅ XB** and **1 ⋅ HB** in the same solvent system. ^1^H NMR experiments were conducted by adding aliquots of halide anions as their tetrabutylammonium (TBA) salts. Upon addition of halide anions to a solution of **1 ⋅ XB**, the most notable perturbation in chemical shift was observed for the internal benzene proton (a), which is consistent with anion binding in the bis‐triazole cleft, and it was this resonance that was monitored as a function of anion concentration. Bindfit^[46]^ analysis of the titration data, resulting from monitoring the proton (a) resonance as a function of anion concentration, determined 1 : 1 stoichiometric halide anion association constants (Table 1).

**Table 1:** Anion binding constants for **1 ⋅ XB** and **1 ⋅ HB** for a variety of halides. (CDCl_3_ : CD_3_CN 1 : 1 (v/v), 500 MHz, 298 K).

	Anion association constant [*K_a_*, M^‐1^]^[a]^	
Anion^[b]^	**1 ⋅ XB**	**1 ⋅ HB**
Cl^−^	85	NB
Br^−^	120	NB
I^−^	138	NB

[a] Determined from Bindfit analysis, monitoring internal benzene proton a. Errors <10% [b] Added as their TBA salts. NB=No binding.

Inspection of the anion binding constants in Table 1 reveals a modest preference of **1 ⋅ XB** for the heavier halides over chloride. By stark contrast, analogous halide anion titrations with **1 ⋅ HB** revealed no significant chemical shift perturbations, indicating no measurable anion binding affinity in the 1 : 1 CD_3_CN/CDCl_3_ (v/v) solvent mixture. This again highlights the often observed superior anion binding capability of XB receptors over HB receptor analogues.[^47^, ^48^, ^49^]

### Ion‐pair Binding Studies

The ion‐pair binding capabilities of **1 ⋅ XB** and **1 ⋅ HB** were investigated by ^1^H NMR titration experiments by adding aliquots of TBA halide salts to an equimolar 1 : 1 CD_3_CN/CDCl_3_ solution of **1 ⋅ XB** and MClO_4_ (M=Na or K). The perchlorate salts were selected as the ClO_4_^−^ anion was expected to be non‐coordinating, which was confirmed by inspection of the ^1^H NMR spectrum of **1 ⋅ XB** with equimolar KClO_4_ in CDCl_3_, which showed no perturbation of the signal arising from proton (a), which is intimately associated with the XB anion binding site, relative to the free receptor (Figure S.15).^[50]^ The anion induced chemical shift perturbations were monitored and analyzed by Bindfit, which determined 1 : 1 apparent host‐guest binding affinities summarized in Table 2.

**Table 2:** Apparent anion binding constants for the M^+^ complexes of **1 ⋅ XB** and **1 ⋅ HB** for a variety of halides. (CDCl_3_ : CD_3_CN 1 : 1 (v/v), 500 MHz, 298 K).

	Apparent anion association constant [*K_a_*, M^‐1^]^[a]^			
Anion^[b]^	**1 ⋅ XB‐K^+^**	**1 ⋅ HB‐K^+^**	**1 ⋅ XB‐Na^+^**	**1 ⋅ XB‐2Na^+^**
Cl^−^	>10^5^	1270	[c]	[c]
Br^−^	>10^5^	997	1710	2680
I^−^	>10^5^	1580	2310	3960

[a] Apparent binding constants determined from Bindfit analysis, monitoring internal benzene proton a. Errors <10% [b] Added as their TBA salts. [c] Salt recombination.

Ion‐pair titration experiments conducted with **1 ⋅ XB** in the presence of equimolar KClO_4_ elicited large shifts in the receptor's proton resonances proximal to the XB anion binding cleft, and notably the halide affinities were universally determined to be >10^5^ M^−1^, corresponding to an increase in *K_a_* of at least two orders of magnitude. The origin of this cooperativity is attributed to three factors: (i) proximal electrostatic attraction between potassium cation and halide anion; (ii) pseudo‐macrocyclic pre‐organization of the bis‐iodotriazole anion binding cleft upon formation of the potassium cation sandwich complex, i. e. an allosteric effect; (iii) potential K^+^ bis‐B15C5 complex formation induced polarization of the C−I bond resulting in a stronger σ‐hole⋅⋅⋅anion interaction.

Analogous ion‐pair titration experiments conducted with **1 ⋅ HB**‐K^+^, resulted in *K_a_* values in the range 997 to 1580 M^−1^ – a total ‘switch‐on’ of anion recognition relative to the neutral HB receptor, which showed no measurable anion affinity. This presumably reflects the inherent electrostatic attraction between the cationic complex and halide anions, in addition to the K^+^ sandwich complex pre‐organization of the HB anion binding site. Importantly, comparison between **1 ⋅ XB** and **1 ⋅ HB** clearly demonstrates the role of XB interactions in driving the exceptionally high cooperativity observed for **1 ⋅ XB**.

**Figure 3:**
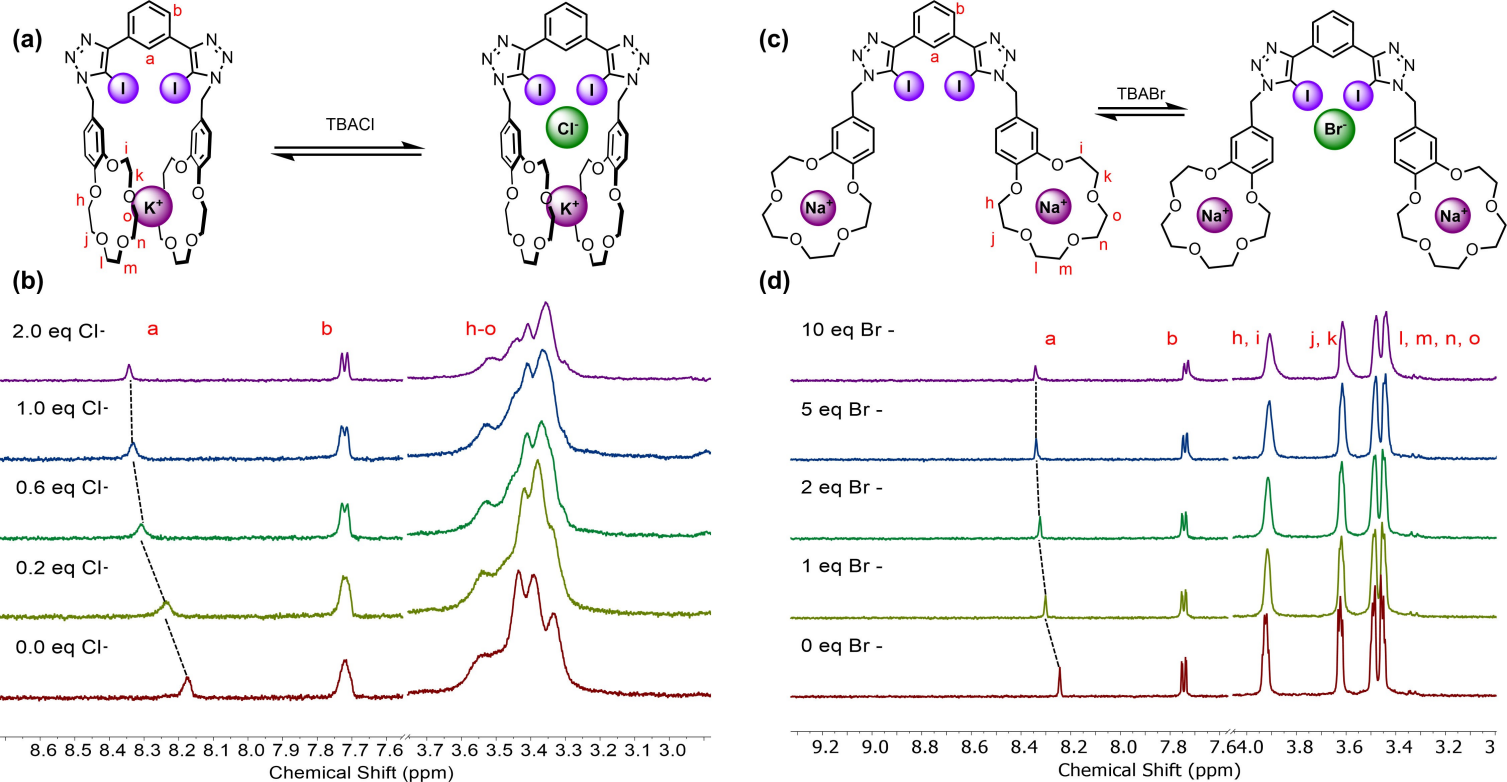
(a) Proposed anion binding mode of **1 ⋅ XB**‐K^+^. (b) Stacked, truncated ^1^H NMR spectra of **1 ⋅ XB**‐K^+^ upon addition of TBACl. (c) Proposed anion binding mode of **1 ⋅ XB**‐Na^+^. (d) Stacked, truncated ^1^H NMR spectra of **1 ⋅ XB**‐Na^+^ upon addition of TBABr.

Saliently, the diagnostic spectroscopic features of the intramolecular K^+^ bis‐B15C5 sandwich complex persist upon the addition of halide anions for both pre‐complexed **1 ⋅ XB**‐K^+^ and **1 ⋅ HB**‐K^+^, indicating that halide anion binding occurs concomitantly with K^+^ complexation (Figure 3b). It is noteworthy that both heteroditopic receptors mediate the stabilization of even the KCl ion‐pair, which is particularly impressive given the high lattice enthalpy of the alkali metal halide salt. This can be ascribed to potassium cation bis‐B15C5 sandwich complex pre‐organization of the respective receptor's XB bis‐iodotriazole (Figure 3a) and HB bis‐prototriazole anion binding clefts. The visual similarity of the **1 ⋅ XB**‐K^+^ TBAI titration spectra to the spectrum of **1 ⋅ XB** complexed with equimolar KI confirmed the non‐coordinating nature of the counter‐ions, TBA^+^ and ClO_4_^−^ (Figure S.13).

In order to elucidate the cooperative role of pre‐organising the bis‐iodotriazole cleft, conferred by intramolecular K^+^ sandwich complex formation, over solely electrostatic attraction between the co‐bound ion‐pair partners, analogous ^1^H NMR ion‐pair binding studies were conducted with sodium halide salts. In the presence of equimolar Na^+^, the apparent bromide and iodide *K_a_* values for **1 ⋅ XB**‐Na^+^ were determined to be 1710 M^−1^ and 2310 M^−1^ respectively, corresponding to an increase in anion affinity by a factor of 15 compared with free **1 ⋅ XB**, consistent with the anticipated proximal electrostatic‐based enhancement of anion binding. However, the addition of Cl^−^ led to salt recombination and precipitation of NaCl, reflecting the higher lattice enthalpy of NaCl relative to NaBr and NaI, which further demonstrates the importance of the intramolecular sandwich complex formation and associated allosteric effect in concomitant KCl ion‐pair binding.

The much larger enhancement in *K_a_* value upon K^+^ complexation relative to Na^+^ indicates, assuming the electrostatic‐based enhancements are similar for each complex, the pre‐organization of the XB binding cleft afforded by the formation of a K^+^ bis‐B15C5 sandwich complex serves as a much more potent positive cooperativity mechanism in this receptor system. Indeed, the significance of allosteric effects over electrostatic effects is further underscored by experiments conducted with **1 ⋅ XB** in the presence of two equivalents of NaClO_4_. Conceivably, the saturation of both B15C5 units of **1 ⋅ XB** with sodium cations should afford a di‐cationic complex and would therefore be expected to further increase halide binding potency. Inspection of Table 2 does indeed reveal an increase in both *K_a_* (Br^−^) and *K_a_* (I^−^) values upon increasing Na^+^ equivalents, to 2680 M^−1^ and 3960 M^−1^, but crucially are still of significantly smaller magnitude than the *K_a_* values obtained by pre‐complexation with one equivalent of K^+^ (*K_a_* >10^5^ M^−1^).

Encouraged by these results, we sought to investigate other alkali metal cations known to form sandwich type complexes with B15C5, such as rubidium.^[51]^ Initial complexation studies with RbClO_4_ revealed diagnostic changes in the crown‐ether methylene region indicative of intramolecular sandwich complex formation, comparable to that observed with K^+^. Analogous ion‐pair binding titration experiments, conducted in the presence of 1 equivalent of RbClO_4_, showed very strong anion binding, with binding constants >10^5^ M^−1^ for all halide anions, including chloride, once again highlighting the potency of the allosteric pre‐organisation of the XB bis‐iodotriazole cleft as a cooperativity mechanism.

**Figure 4:**
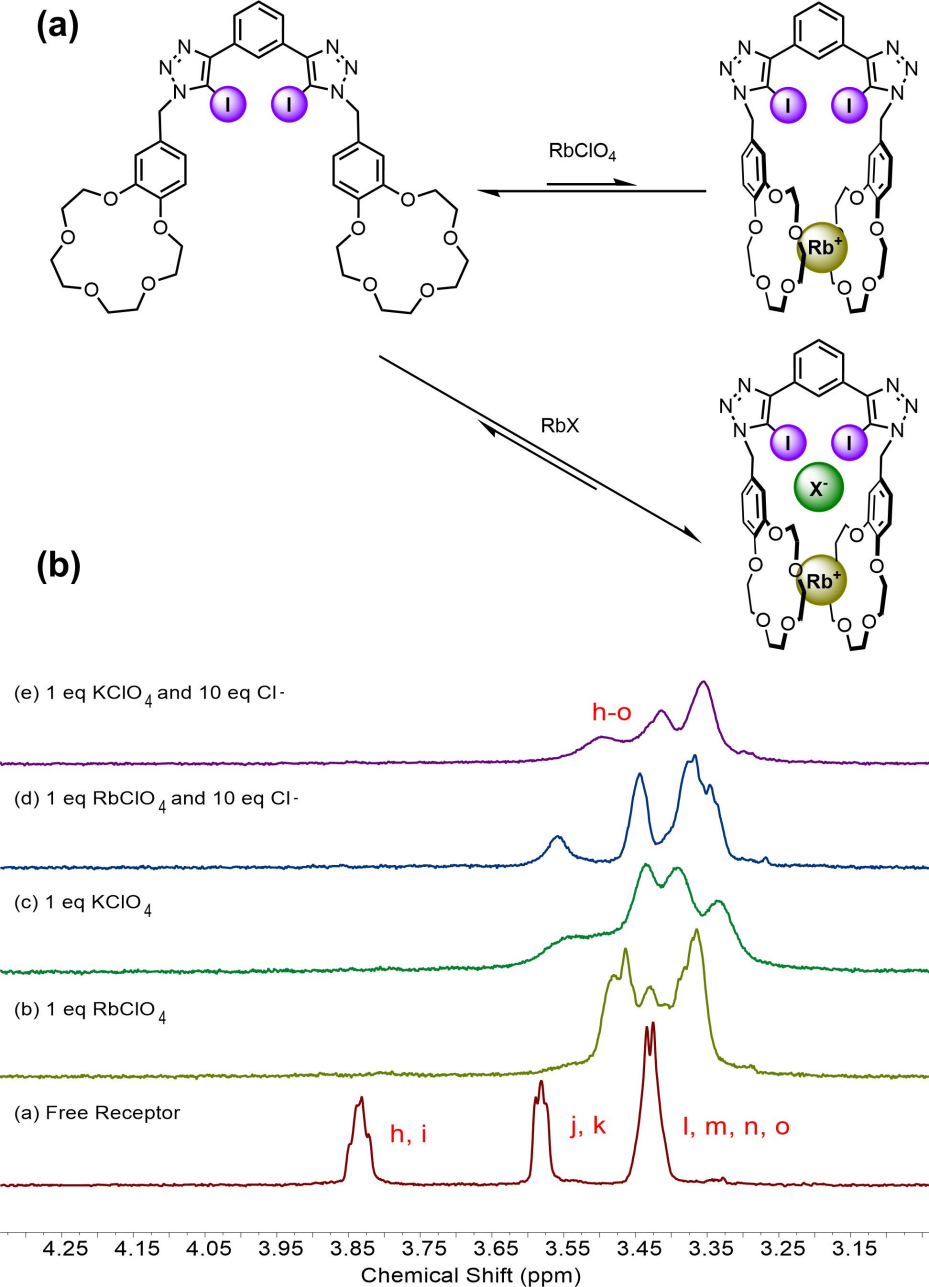
(a) Proposed strengthening of Rb^+^ sandwich complex upon anion binding (b) Stacked, truncated ^1^H NMR spectra of **1 ⋅ XB** with various ion‐pairs, illustrating changes to crown‐ether methylene resonances.

Relative to the ion‐pair titration experiments with **1 ⋅ XB**‐K^+^, more significant changes in the crown‐ether methylene resonances were observed over the course of the ion‐pair titrations with **1 ⋅ XB**‐Rb^+^ (Figure 4b). It is important to note that the spectroscopic perturbations are still consistent with simultaneous rubidium cation and halide anion binding as for the ion‐pair titration experiments with **1 ⋅ XB**‐K^+^. However, the nature of these crown‐ether based chemical shift changes are consistent with an anion‐induced cooperative strengthening of the Rb^+^ bis‐B15C5 sandwich complex, which is evidenced by further broadening of the crown‐ether methylene resonances of **1 ⋅ XB**‐Rb^+^ upon halide addition and their increasing resemblance to those of **1 ⋅ XB**‐K^+^ at the end of the ion‐pair titration. Interestingly, this suggests a synergistic cooperative effect wherein the binding of an anion enhances also the complex's affinity for a cation.

## Conclusion

The mechanisms giving rise to cooperativity in heteroditopic ion‐pair receptors are often difficult to disentangle, which has inhibited their considered application in heteroditopic receptor design. Through the development of a novel XB ion‐pair receptor consisting of a 1,3‐bis‐iodo‐triazole benzene XB anion binding site, flexibly appended via a methylene linker with two benzo‐15‐crown‐5 (B15C5) cation binding moieties, and its HB analogue, this study has provided a systematic insight into the relative contributions of electrostatics, and allostery and halogen bonding in alkali metal halide ion‐pair recognition. The delineation of cooperativity mechanisms was achieved by exploiting the contrasting alkali metal cation binding modes of **1 ⋅ XB**, which in turn are driven by the cation‐radius‐dependent binding characteristics of B15C5. These cation binding modes were established via ^1^H NMR titrations, followed by extensive ion‐pair binding studies which also illustrated the remarkable potency of XB interactions in the context of ion‐pair recognition.

Crucially, the overwhelming importance of employing a positive allosteric effect in enhancing cooperativity was highlighted, with halide anion binding affinity notably increased by a factor of at least 700 in the cases of **1 ⋅ XB**‐K^+^ or **1 ⋅ XB**‐Rb^+^, when both allosteric pre‐organisation via intramolecular alkali metal cation bis‐B15C5 sandwich complexation and electrostatic mechanisms are employed. This compares to a halide anion enhancement affinity factor of just 15 for **1 ⋅ XB**‐Na^+^, where only an electrostatic contribution to cooperativity is possible.

Hence, we have shown electrostatic attraction, judiciously incorporated allosteric design elements and polarised XB mediated anion recognition are important design features to consider and combine for potentially maximising positive cooperativity in future heteroditopic ion‐pair receptor development.

## Conflict of interest

The authors declare no conflict of interest.

1

## Supporting information

As a service to our authors and readers, this journal provides supporting information supplied by the authors. Such materials are peer reviewed and may be re‐organized for online delivery, but are not copy‐edited or typeset. Technical support issues arising from supporting information (other than missing files) should be addressed to the authors.

Supporting Information

## Data Availability

The data that support the findings of this study are available in the supplementary material of this article.
