# Supplementary material for: Allosteric and Electrostatic Cooperativity in a Heteroditopic Halogen Bonding Receptor System
Source: Chem Asian J. 2022 Dec 28;18(3):e202201170. doi: 10.1002/asia.202201170 (PMC10107604; doi:10.1002/asia.202201170)
Supplement: Supplementary file 1 — Supporting Information [file ASIA-18-0-s001.pdf]

CHEMISTRY
AN ASIAN JOURNAL

Supporting Information

Allosteric and Electrostatic Cooperativity in a Heteroditopic Halogen Bonding Receptor System

Andrew J. Taylor[+], Andrew Docker[+], and Paul D. Beer*This manuscript is part of a special collection on Halogen Bonding.© 2022 The Authors. Chemistry - An Asian Journal published by Wiley-VCH GmbH. This is an open access article under the terms of the Creative Commons Attribution License, which permits use, distribution and reproduction in any medium, provided the original work is properly cited.

Contents

1. General Procedures 1
Chemicals and Solvents 1
Instrumental Methods 2
2. Titration Protocols 2
Titration Procedure 2
Data Fitting 2
3. [1]NMR Spectra of Ion-pair Binding Studies 3
Cation Titration Spectra 3
Anion Titration Spectra 5
Direct Complexation Studies 10
4. Synthesis of Novel Compounds 12
4'-Azidomethylbenzo-15-crown-5 (5) 12
General Synthetic Procedure for XB and HB Ion-pair hosts 12
5. Characterisation of Novel Compounds 12
4'-Azidomethylbenzo-15-crown-5 (5) 12
1•XB 16
1•HB 19

1. General Procedures

Chemicals and Solvents

All solvents and reagents were purchased form commercial suppliers and were used without further purification unless otherwise stated. Anhydrous solvents were obtained by purging with nitrogen and then passing though an MBraun MPSP-800 column. H_2O was de-ionised and micro filtered using a Milli-Q® Millipore machine. TBTA, TBA salts and [Cu(MeCN)4]PF6 were stored

under vacuum over phosphorus pentoxide prior to use. Triethylamine was distilled from and stored over potassium hydroxide. Column chromatography was carried out on Merck® silica gel 60 under a positive pressure of nitrogen. Where mixtures of solvents were used, ratios have been reported by volume (v/v).

Instrumental Methods

Routine NMR were recorded on Bruker AVIII HD 400 and Bruker AVIII 500 spectrometers. Chemical shift (δ) values are quoted in parts per million (ppm) and are relative to the residual solvent peak. Low-resolution ESI mass spectra were recorded on a Waters LCT premier spectrometer, whereas high-resolution mass spectra were recorded on a Bruker µTOF spectrometer to 4 decimal places.

2. Titration Protocols

Titration Procedure

^{1}H NMR titration experiments were conducted on and Bruker AVIII 500 spectrometers at 298K. Host samples were prepared in 0.5 mL volume and generally at 1.0 mM concentration. Solutions of anionic guest species (50 mM) as their TBA salts were added to the host samples as aliquots in increments of 0.0, 0.2, 0.4, 0.6, 0.8, 1.0, 1.2, 1.4, 1.6, 1.8, 2.0, 2.5, 3.0, 4.0, 5.0, 7.0 and 10.0 equivalents, except in the case of titrations involving neutral **1•HB**, which did not show a response to anion addition, and in which case increments of 0.0, 0.2, 0.4, 0.6, 0.8, 1.0 and 10.0 equivalents were used. Solutions of cationic guest species (50 mM) as their BAr^F_4 salts were added to the host samples as aliquots in increments of 0.0, 0.1, 0.2, 0.3, 0.4, 0.5, 0.6, 0.7, 0.8, 0.9, 1.0, 1.2, 1.4, 1.6, 1.8, 2.0, 3.0, and 4.0 equivalents. Samples were thoroughly shaken after the addition of each aliquot before recording their spectra. Unless specified, the bound and unbound species were found to be in fast exchange on the NMR timescale.

Data Fitting

Binding stoichiometries were obtained by visual analysis of the titration data. Association constants were determined using Bindfit, in which the experimental titration data was fitted to a 1:1 host : guest binding model. The standard error of measurement in the binding constants provided by the Bindfit model were typically found to be less than 10% unless specified. These errors were considered when comparing similarities and differences between association constants. Possible systematic errors are not considered within this error, however, and only the error associated with the fitting of a binding isotherm to the titration data is accounted for. It is possible, therefore, that error magnitudes are underestimated.

Experimental binding isotherms shown in the text were generated from Bindfit data using OriginPro 2017.

3. [1]NMR Spectra of Ion-pair Binding Studies

Cation Titration Spectra

The full, stacked titration spectra obtained by the addition of $NaBAr^F{}_4$ and $KBAr^F{}_4$ to solutions of **1•XB** and B15C5 are shown.

1•XB $NaBAr^F{}_4$

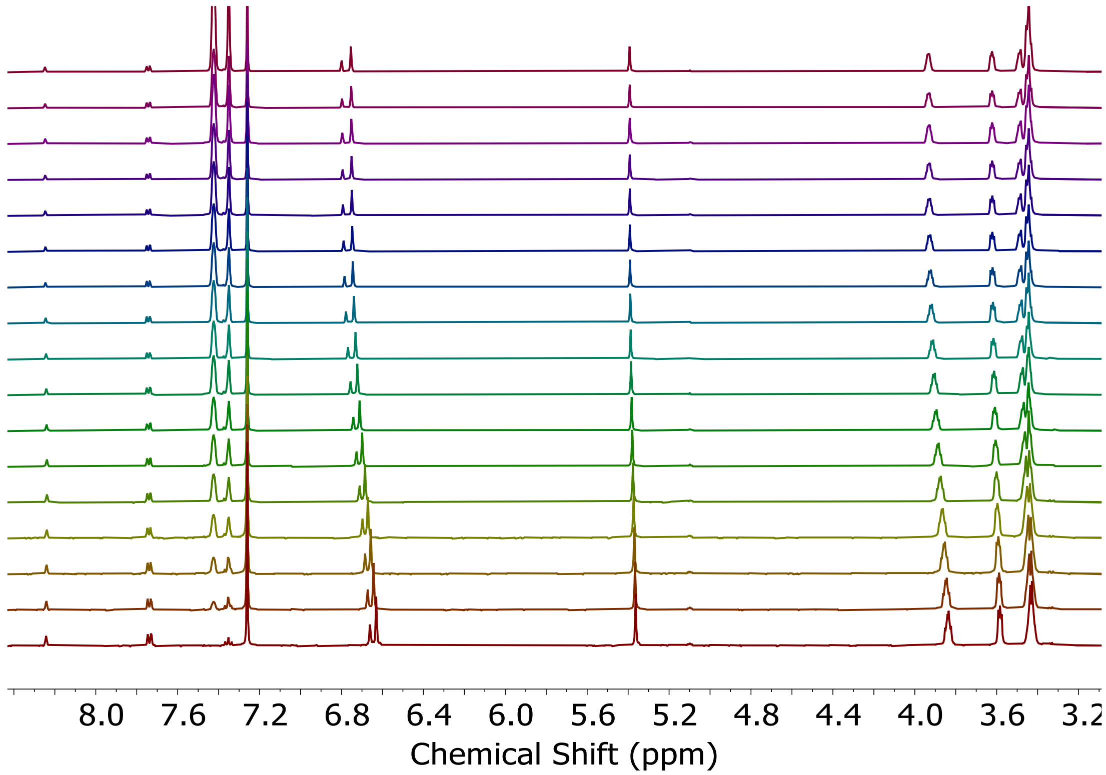

Figure S1 Stacked titration ^1H NMR spectra obtained by the addition of $NaBAr^F{}_4$ to a solution of **1•XB**. (1mM, $CDCl_3$:CD_3CN 1:1 (v/v), 298K).

1•XB KBAr$^F_4$

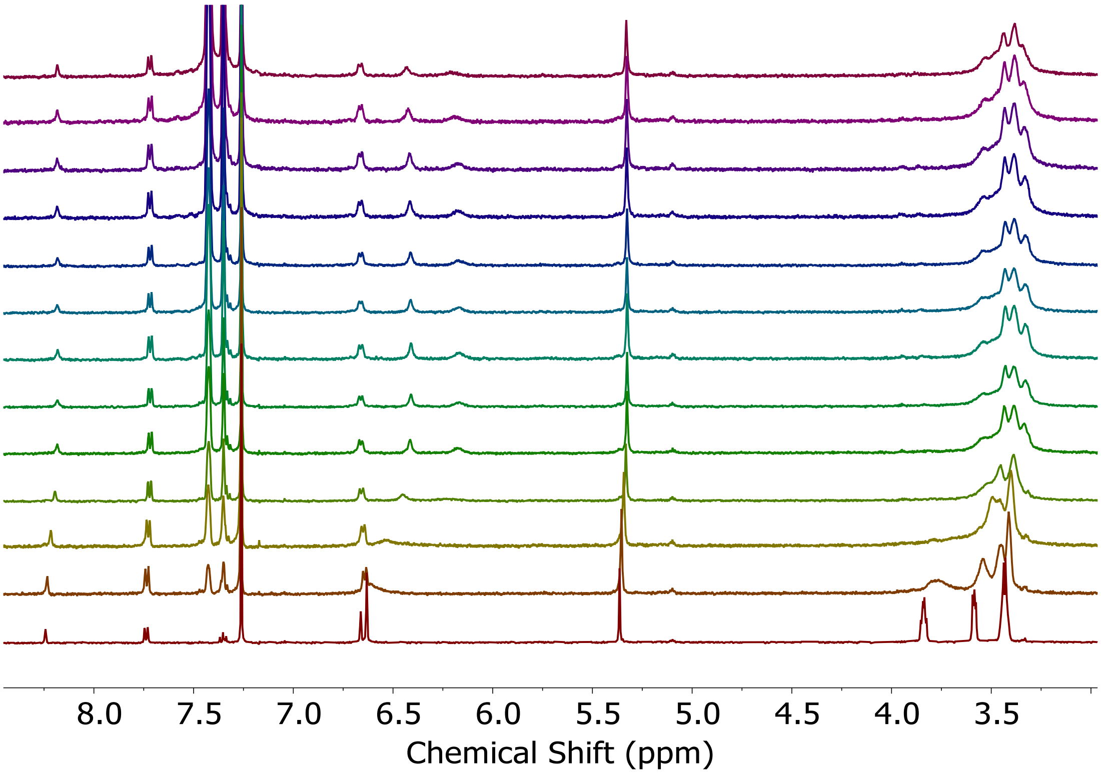

Figure S2 Stacked titration ^{1}H NMR spectra obtained by the addition of KBAr$^F_4$ to a solution of **1•XB**. (1mM, $CDCl_3$:CD_3CN 1:1 (v/v), 298K).

B15C5 NaBAr$^F_4$

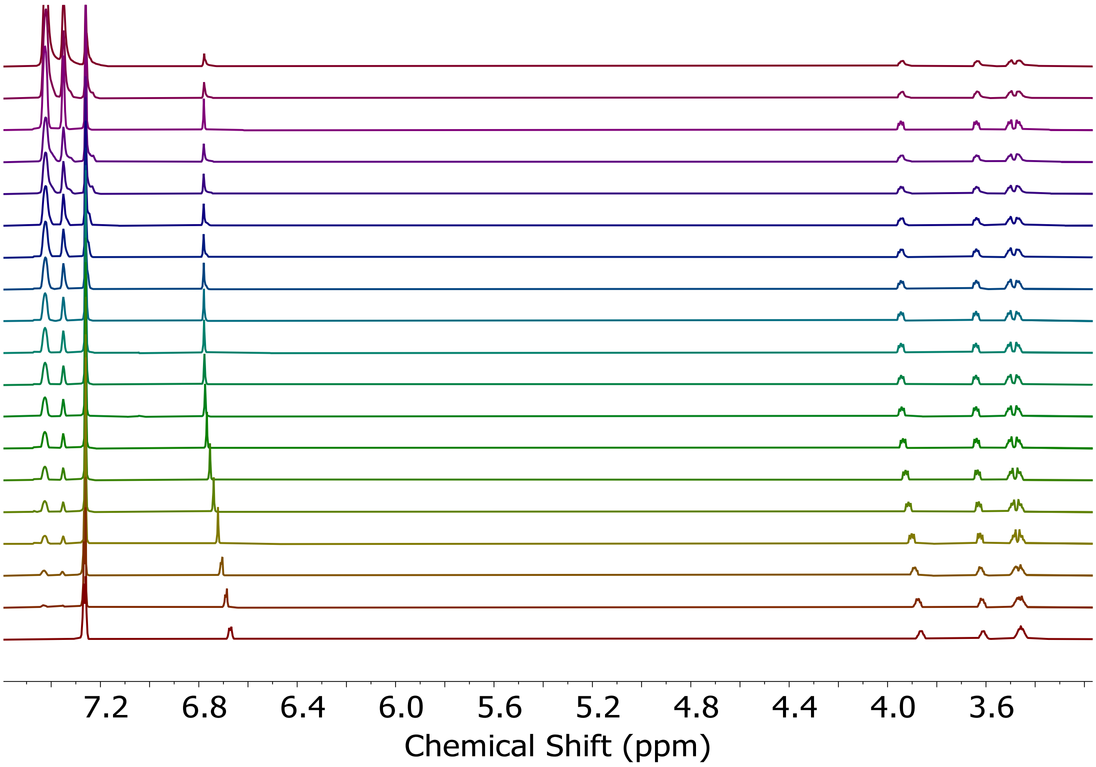

Figure S3 Stacked titration ^{1}H NMR spectra obtained by the addition of NaBAr$^F_4$ to a solution of B15C5. (1mM, $CDCl_3$:CD_3CN 1:1 (v/v), 298K).

B15C5 $KBAr^F_4$

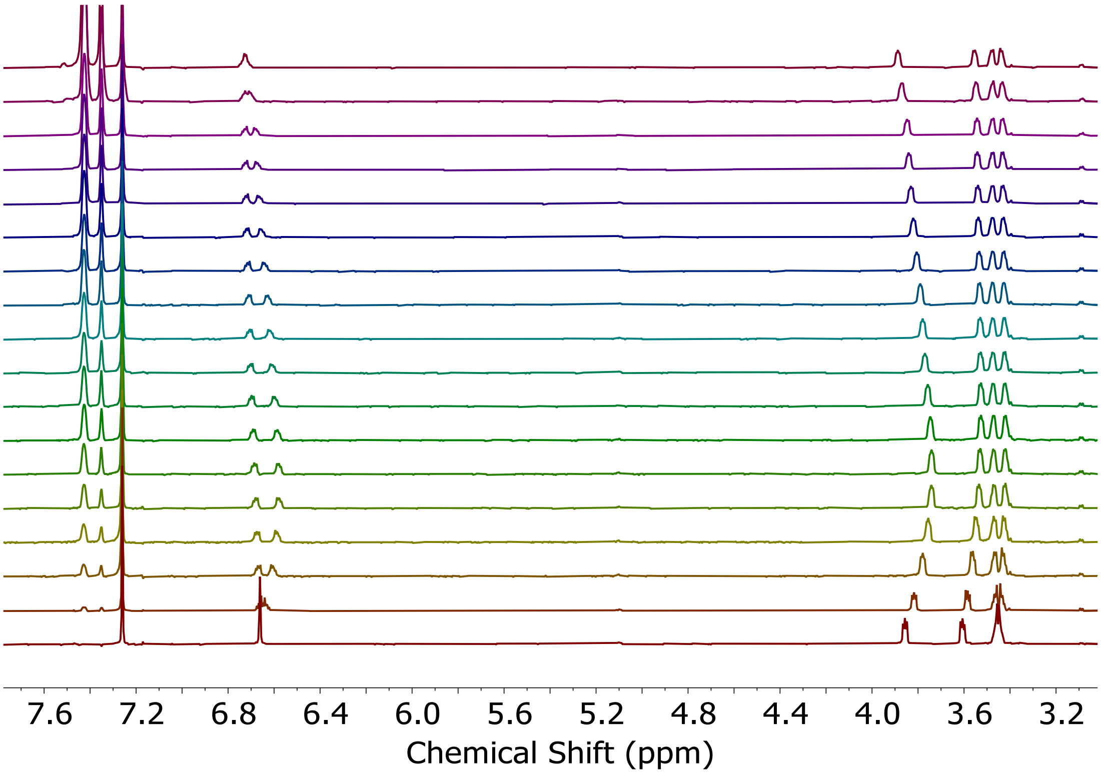

Figure S4 Stacked titration ^{1}H NMR spectra obtained by the addition of $KBAr^F_4$ to a solution of B15C5. (1mM, $CDCl_3$:CD_3CN 1:1 (v/v), 298K).

Anion Titration Spectra

The full, stacked titration spectra obtained by the addition of TBABr to solutions of **1•XB**, **1•XB**-K^+, **1•HB**-K^+, **1•XB**-Na^+, **1•XB**-$2Na^+$, **1•XB**-Rb^+ are shown as representative examples of anion binding to the receptor or receptor-cation complex. The full, stacked titration spectra obtained by the addition of TBACl to solutions of **1•HB** and **1•XB**-Na^+ are also shown as representative examples of no anion binding and salt recombination respectively.

1•XB TBABr

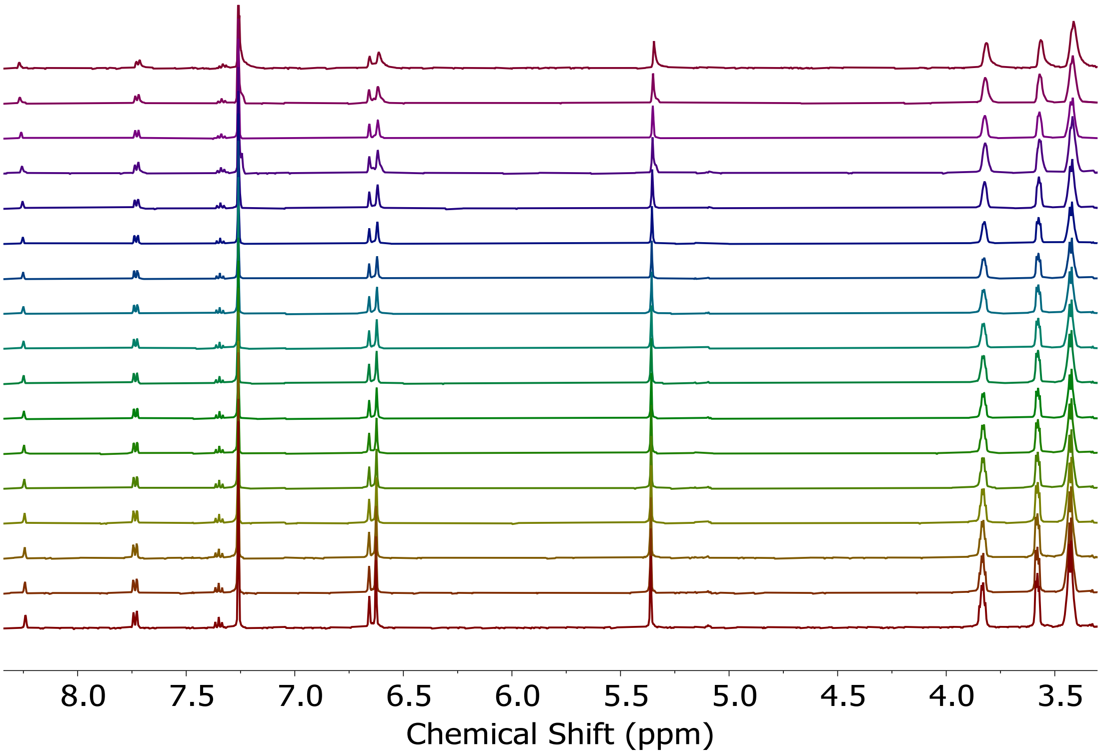

Figure S5 Stacked titration ^{1}H NMR spectra obtained by the addition of TBABr to a solution of **1•XB**. (1mM, $CDCl_3$:CD_3CN 1:1 (v/v), 298K).

1•XB-K^+ TBABr

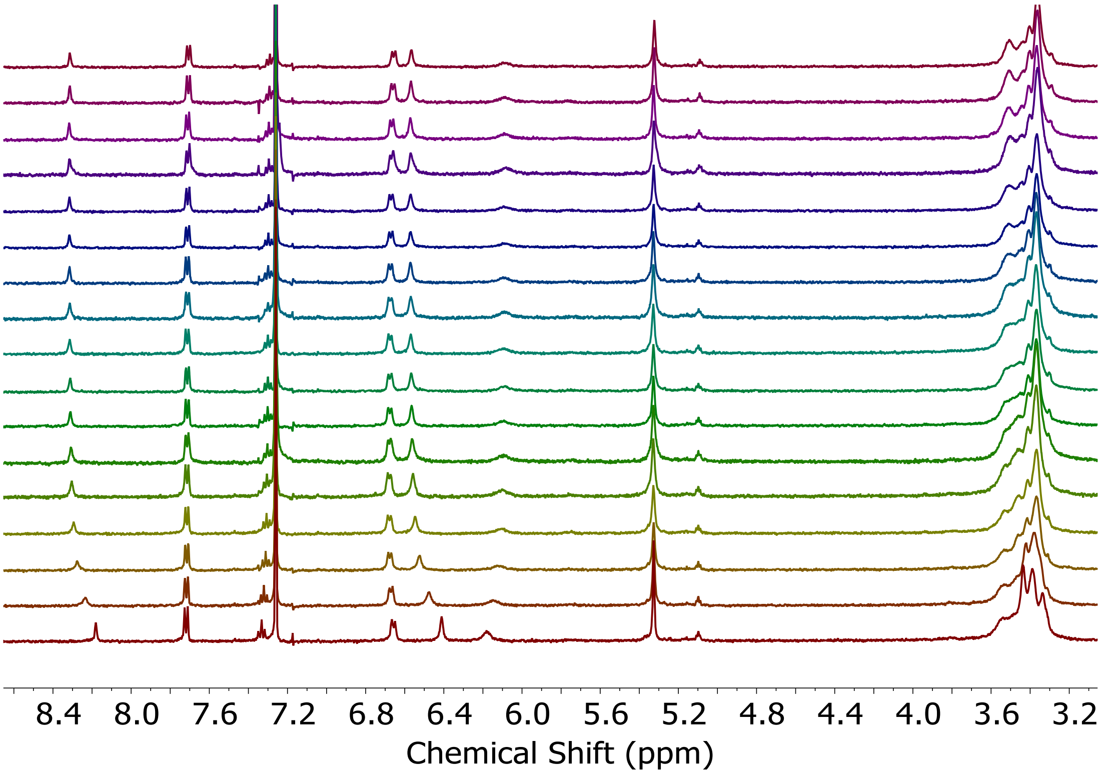

Figure S6 Stacked titration ^{1}H NMR spectra obtained by the addition of TBABr to a solution of **1•XB**-K^+. (1mM, $CDCl_3$:CD_3CN 1:1 (v/v), 298K).

1•**HB**-K⁺ TBABr

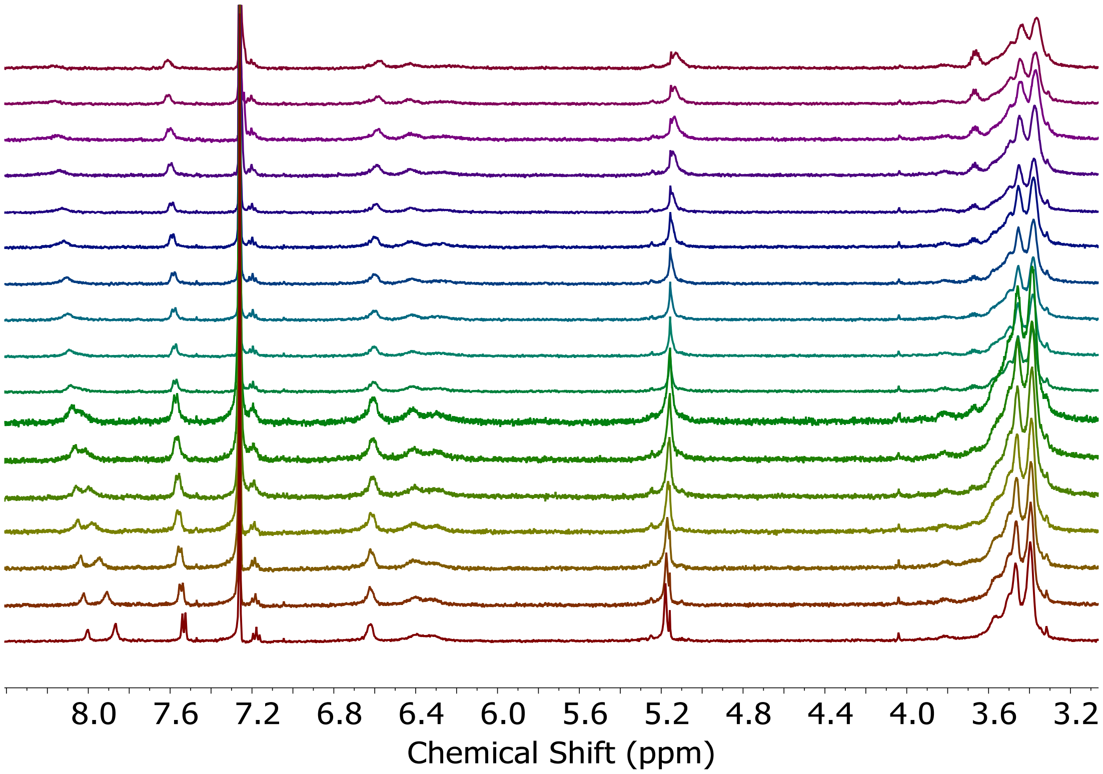

Figure S7 Stacked titration ^{1}H NMR spectra obtained by the addition of TBABr to a solution of **1•HB**-K^{+}. (1mM, $CDCl_3$:CD_3CN 1:1 (v/v), 298K).

1•**XB**-Na⁺ TBABr

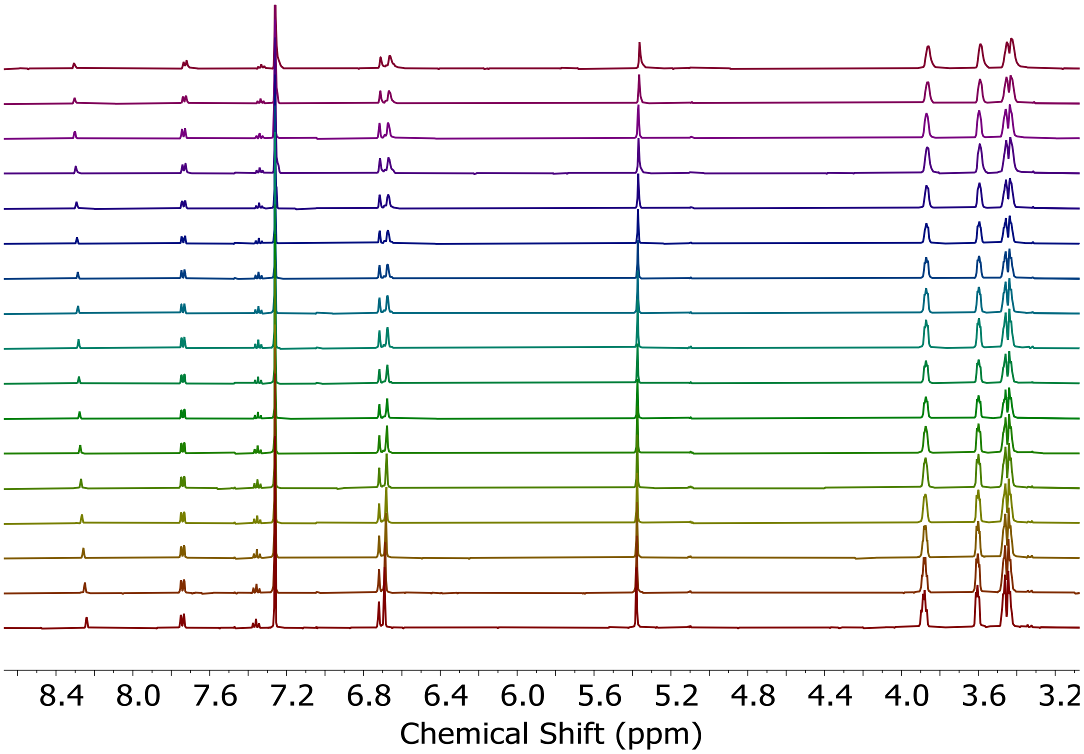

Figure S8 Stacked titration ^{1}H NMR spectra obtained by the addition of TBABr to a solution of **1•XB**-Na^{+}. (1mM, $CDCl_3$:CD_3CN 1:1 (v/v), 298K).

1•XB-2Na⁺ TBABr

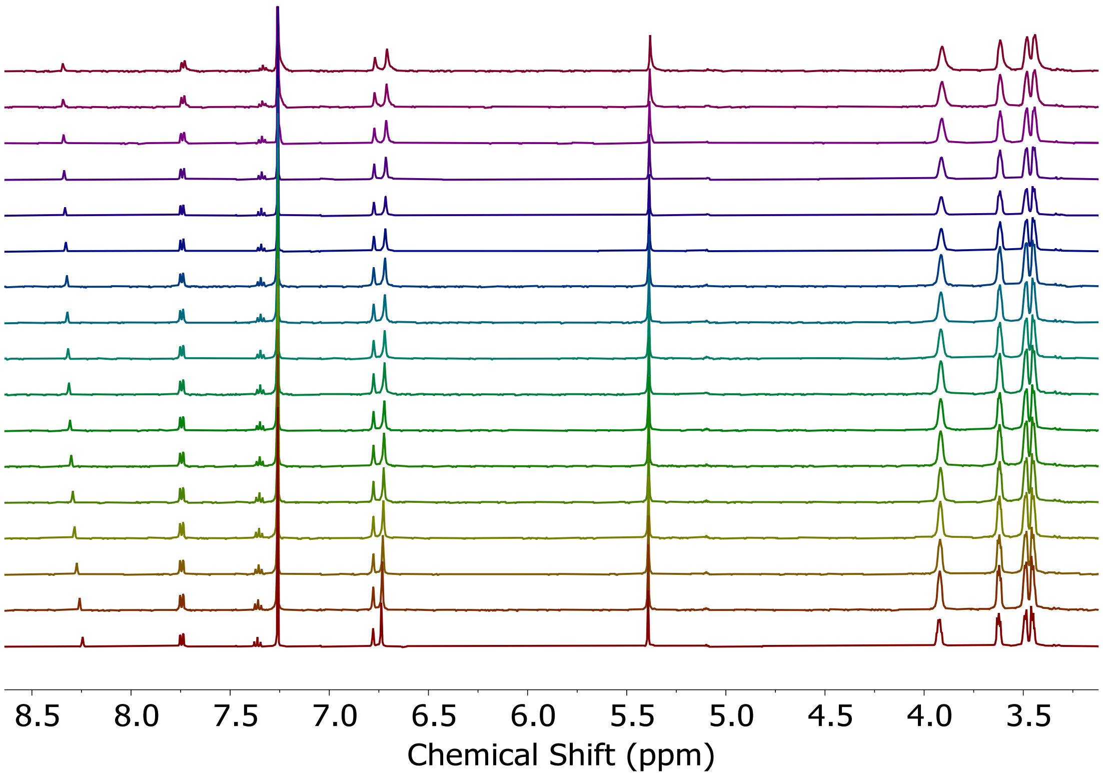

Figure S9 Stacked titration ^{1}H NMR spectra obtained by the addition of TBABr to a solution of **1•XB**-2Na^{+}. (1mM, $CDCl_3$:CD_3CN 1:1 (v/v), 298K).

1•XB-Rb⁺ TBABr

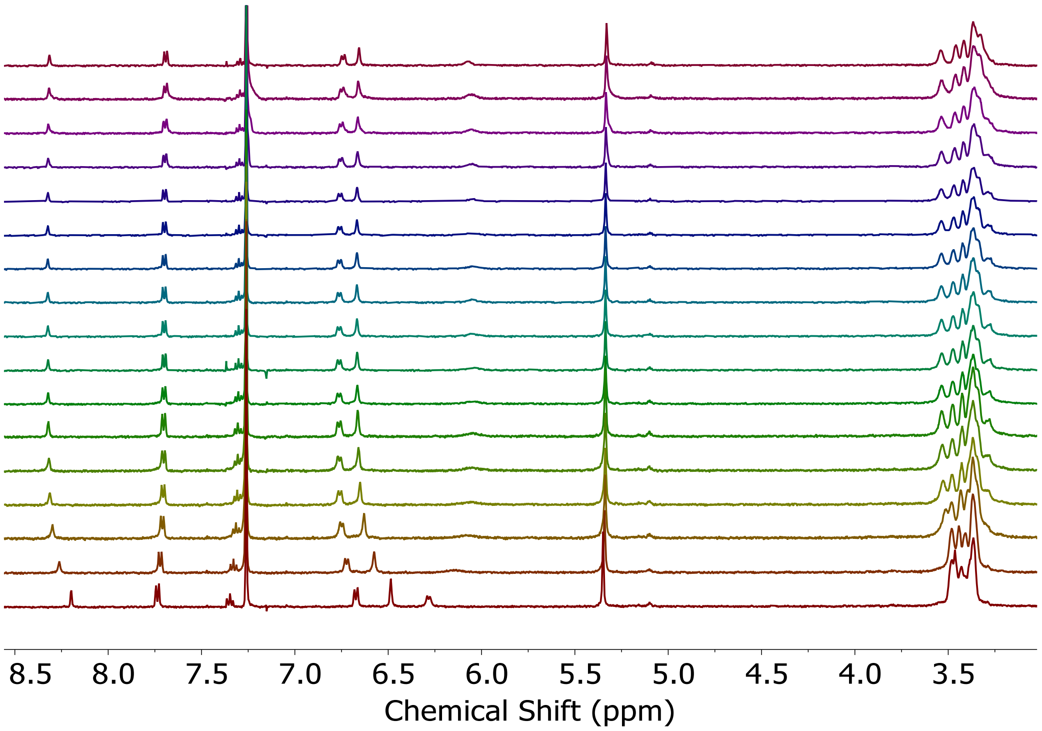

Figure S10 Stacked titration ^{1}H NMR spectra obtained by the addition of TBABr to a solution of **1•XB**-Rb^{+}. (1mM, $CDCl_3$:CD_3CN 1:1 (v/v), 298K).

1•HB TBACl

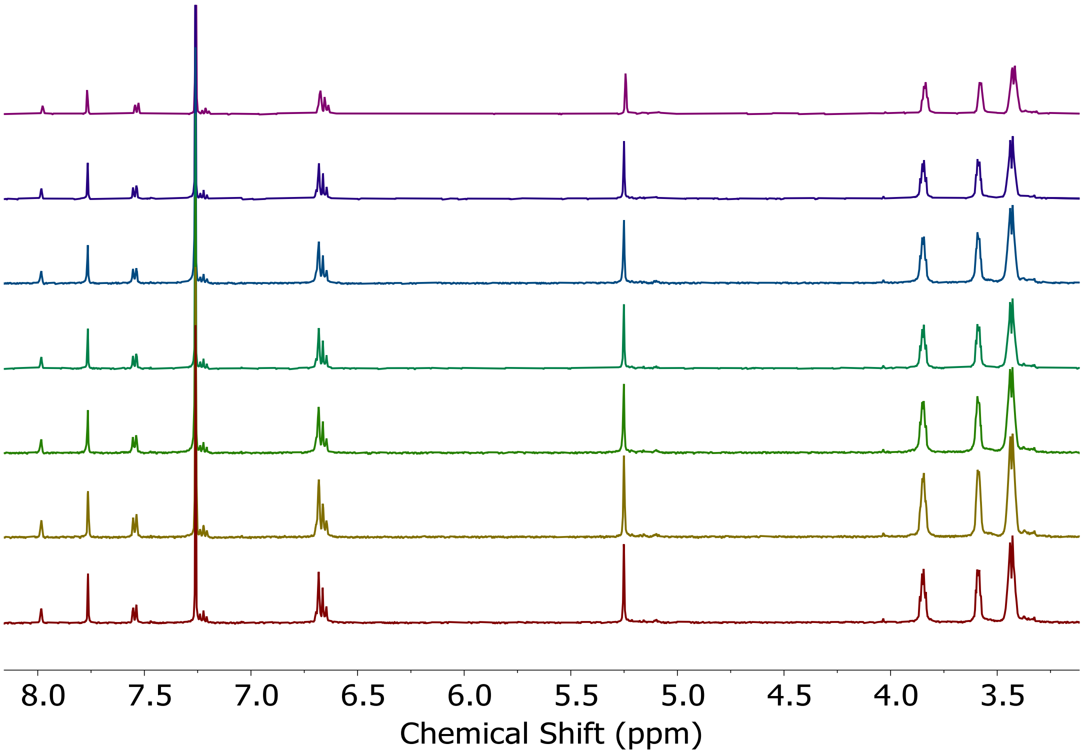

Figure S11 Stacked titration ^{1}H NMR spectra obtained by the addition of TBACl to a solution of **1•HB**. (1mM, $CDCl_3$:CD_3CN 1:1 (v/v), 298K).

1•XB-Na^+ TBACl

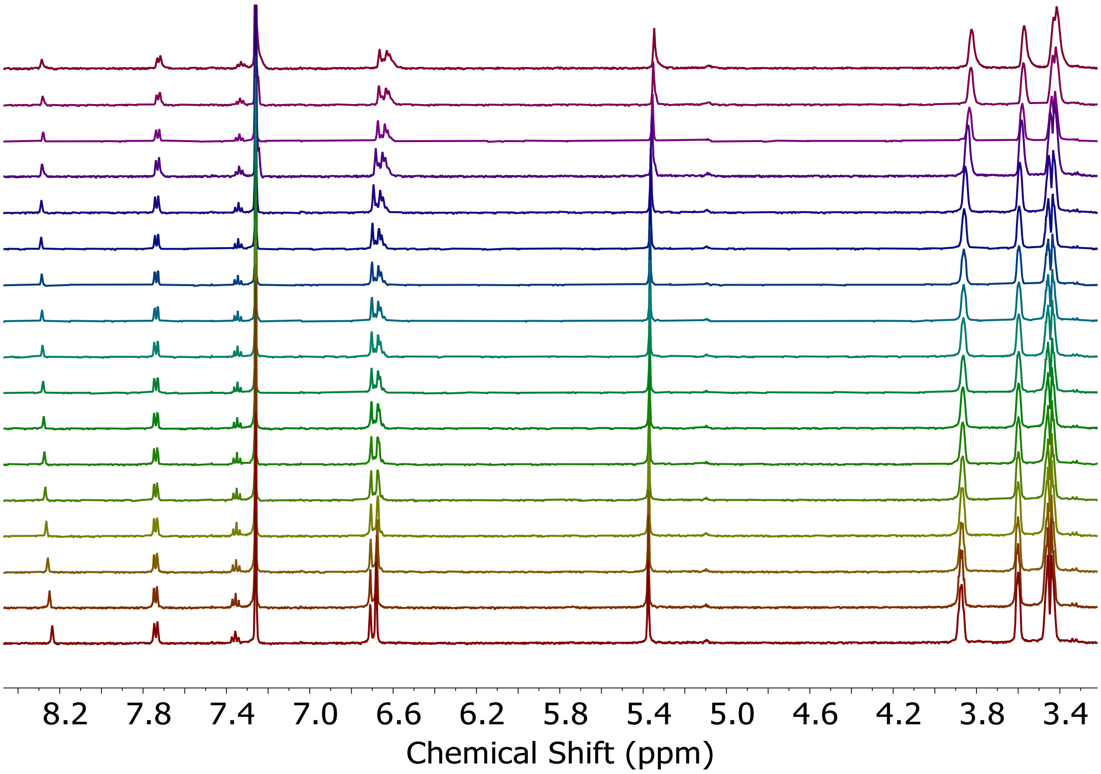

Figure S12 Stacked titration ^{1}H NMR spectra obtained by the addition of TBACl to a solution of **1•XB**-Na^+. (1mM, $CDCl_3$:CD_3CN 1:1 (v/v), 298K).

Direct Complexation Studies

Direct complexation studies of **1•XB** with the iodide salts of potassium and rubidium were performed. Diagnostic changes to the crown-ether methylene protons' signals were seen, indicating the formation of sandwich complexes in all cases. Comparison of titration spectra with these direct complexation spectra provided reassuring evidence that the behaviour observed over the course of the titrations can be ascribed to simultaneous ion-pair binding, which is not affected by the presence of the non-coordinating counter-anions (TBA^+ and ClO_4^-)

1•XB KI

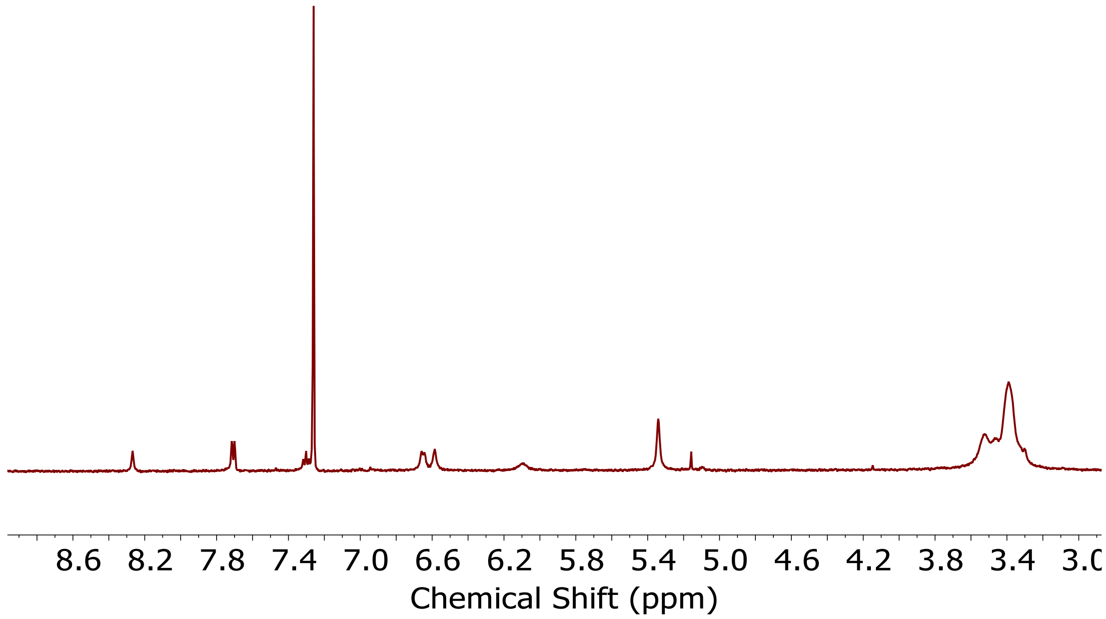

Figure S13 ^{1}H NMR spectra obtained by complexation of 1 equivalent of KI to **1•XB**. (1mM, $CDCl_3$:CD_3CN 1:1 (v/v), 298K).

1•XB RbI

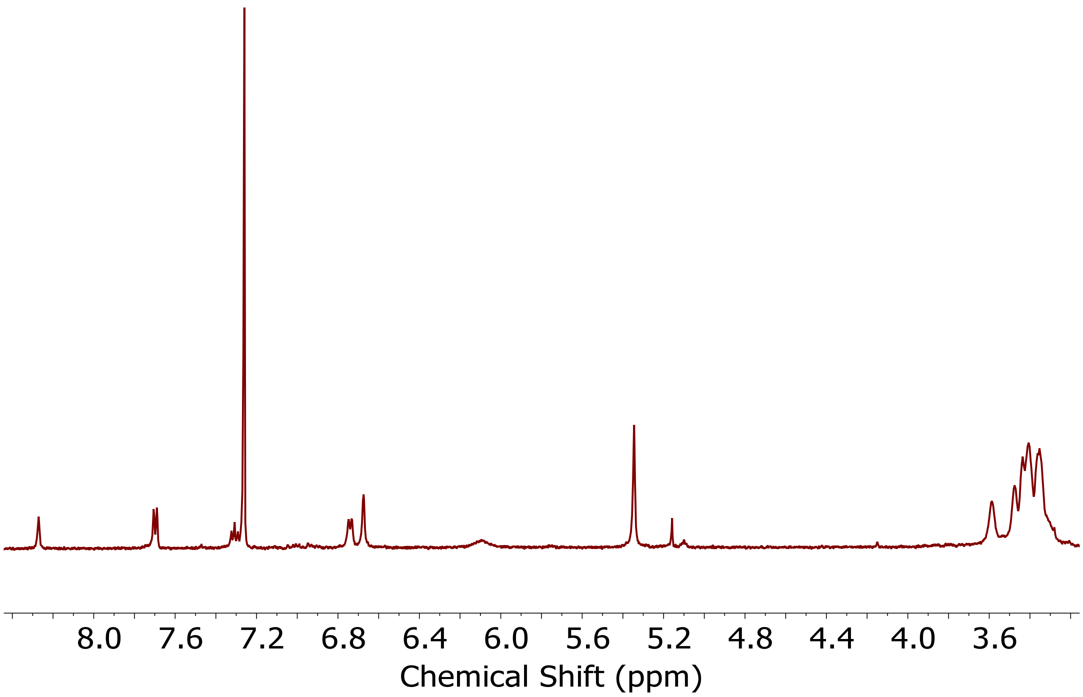

Figure S14 ^{1}H NMR spectra obtained by complexation of 1 equivalent of RbI to **1•XB**. (1mM, $CDCl_3$:CD_3CN 1:1 (v/v), 298K).

1•XB $KClO_4$

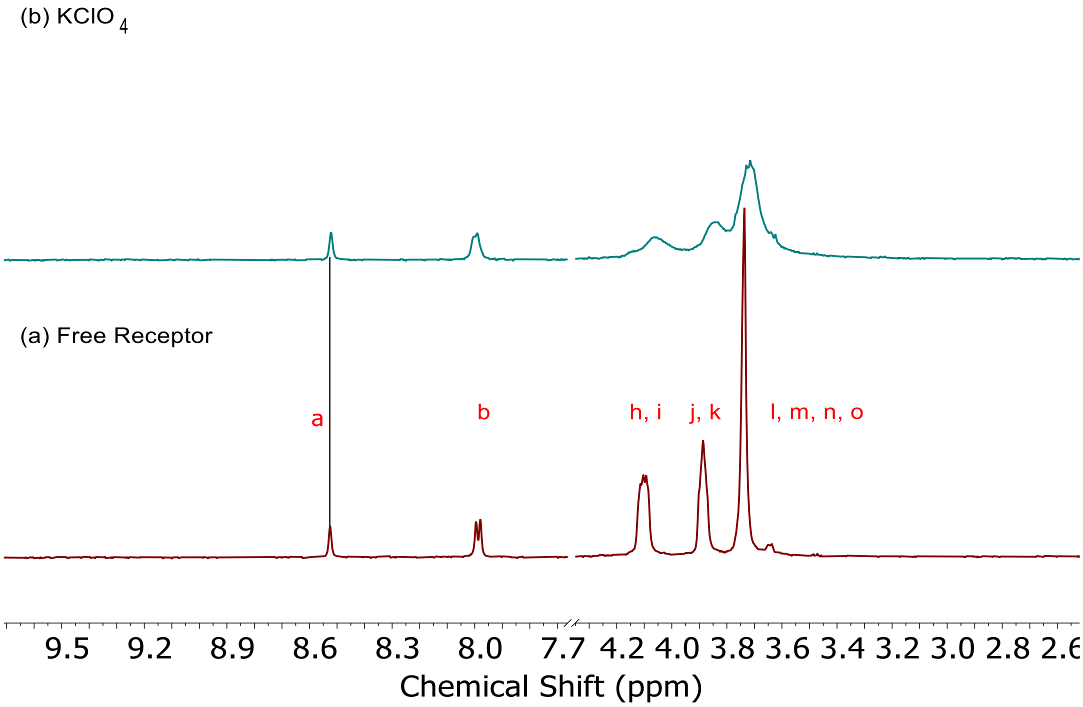

Figure S15 Partial ^{1}H NMR spectra obtained by complexation of 1 equivalent of $KClO_4$ to **1•XB**, indicating the absence of a perturbation in signal (a). (1mM, $CDCl_3$, 298K).

4. Synthesis of Novel Compounds

4'-Azidomethylbenzo-15-crown-5 (5)

4'-Chloromethylbenzo-15-crown-5 (2.20 mmol), NaN_3 (17.7 mmol) and KI (spatula tip) were added to a round-bottomed flask, which was subsequently degassed. Degassed DMF (~5mL) was added and the mixture left to stir overnight at 80°C under N_2, until TLC analysis confirmed full conversion of 4'-chloromethylbenzo-15-crown-5. The mixture was allowed to cool, and the solvent removed *in vacuo*. The crude reaction mixture was partitioned between water (15mL) and EtOAc (25 mL) and the resultant aqueous layer was extracted with EtOAc (10mL) iteratively until no UV-active material remained in the aqueous layer. The combined organic phases were washed once more with water, and then dried over $MgSO_4$, before the solvent was removed *in vacuo*. The resulting yellow oil was cooled to 0°C to induce solidification, affording 4'-azidomethylbenzo-15-crown-5 in quantitative yield.

General Synthetic Procedure for XB and HB Ion-pair hosts

$Cu(MeCN)_4PF_6$ (0.03 mmol) and TBTA (0.03 mmol) were dissolved in degassed CH_2Cl_2:THF 1:1 (*v/v*) (≈ 2 mL) and left to stir for 10 minutes. The requisite 3,5-diethynylbenzene precursor (0.265 mmol) was added to the solution of the copper complex, followed 4'-azidomethylbenzo-15-crown-5 (0.582 mmol). The resultant mixtures were left to stir until TLC analysis confirmed full consumption of the alkyne precursor and mono-triazole intermediates. The crude reaction mixture was diluted with CH_2Cl_2 (10mL) and the organic layer washed with aqueous 0.01 M NH_4OH/EDTA solution (10 mL). The resultant aqueous layer was back extracted with CH_2Cl_2 (2 x 10 mL) and the combined organic phases washed with water before they were dried over $MgSO_4$ and concentrated in vacuo to obtain the crude product mixture. The relevant products were isolated via either alumina-gel chromatography or trituration (*vide infra*).

5. Characterisation of Novel Compounds

4'-Azidomethylbenzo-15-crown-5 (5)

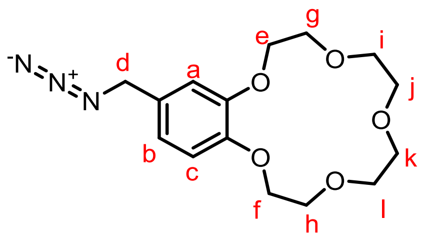

^{1}H NMR (400 MHz, $CDCl_3$) δ = 6.87 – 6.80 (m, $3H_{a,b,c}$), 4.24 (s, $2H_d$), 4.18 – 4.10 (m, $4H_{e,f}$), 3.95 – 3.87 (m, $4H_{g,h}$), 3.81 – 3.72 (m, $8H_{i,j,k,l}$).

^{13}C NMR (101 MHz, $CDCl_3$) δ = 149.49, 149.39, 128.44, 121.51, 114.23, 114.03, 71.27, 70.65, 69.70, 69.22, 69.19, 54.82. (3 peaks missing due to coincident chemical shift).

HRMS (ESI +ve) m/z: 346.1369, ([M+Na]+, $C_{15}H_{21}N_3O_5Na$ requires 346.1373)

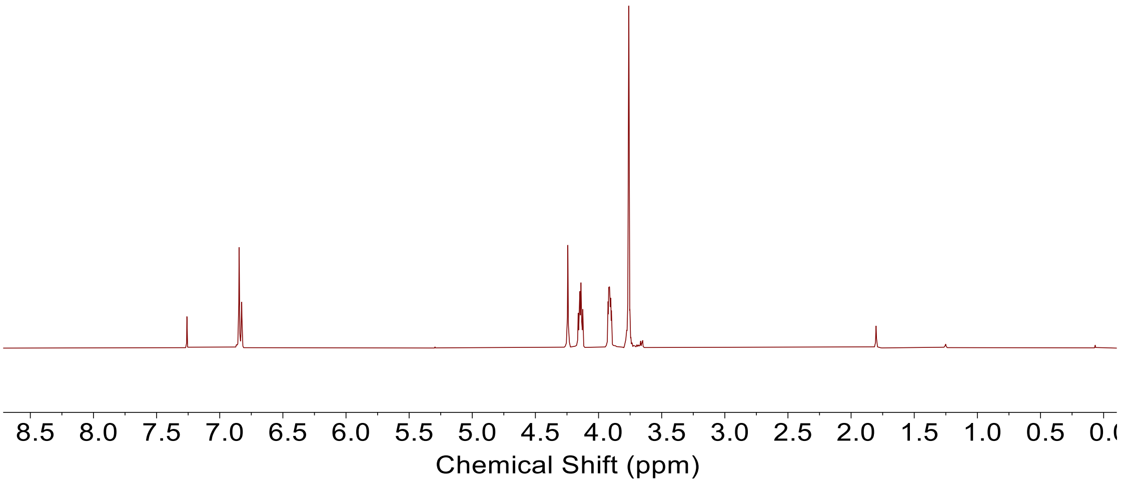

Figure S16 ^{1}H NMR spectrum of 4'-Azidomethylbenzo-15-crown-5 (**5**).

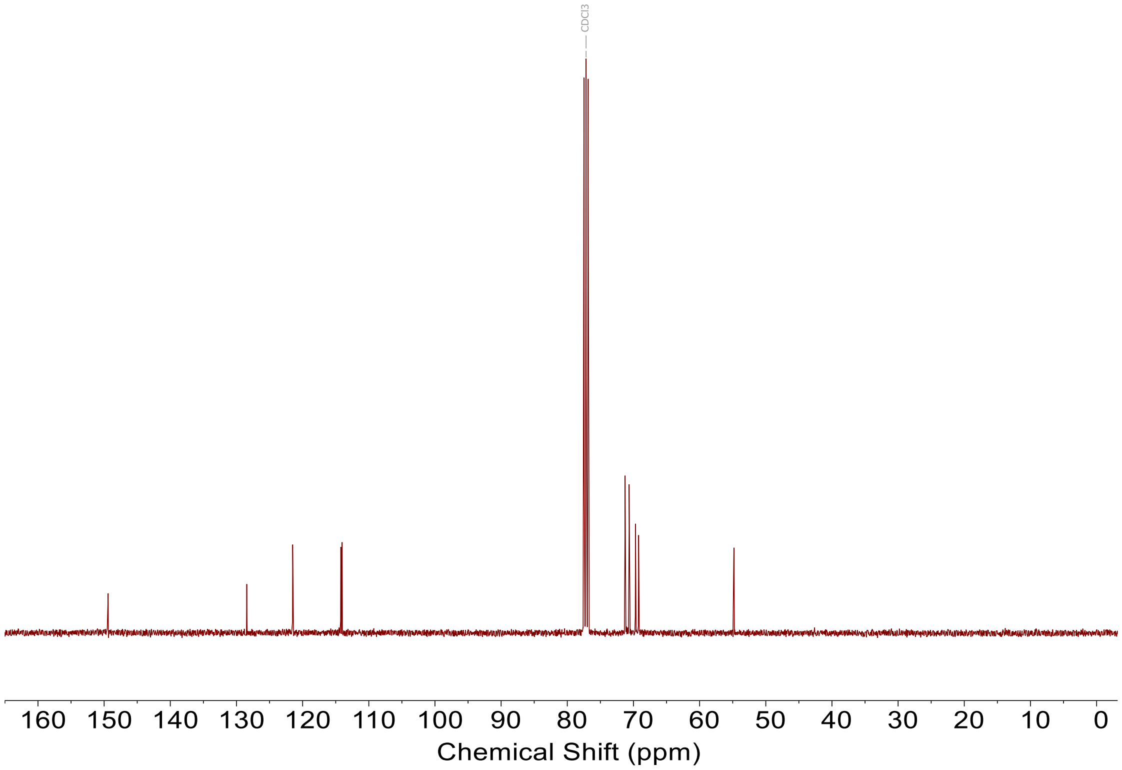

Figure S17 ^{13}C NMR spectrum of 4'-Azidomethylbenzo-15-crown-5 (**5**).

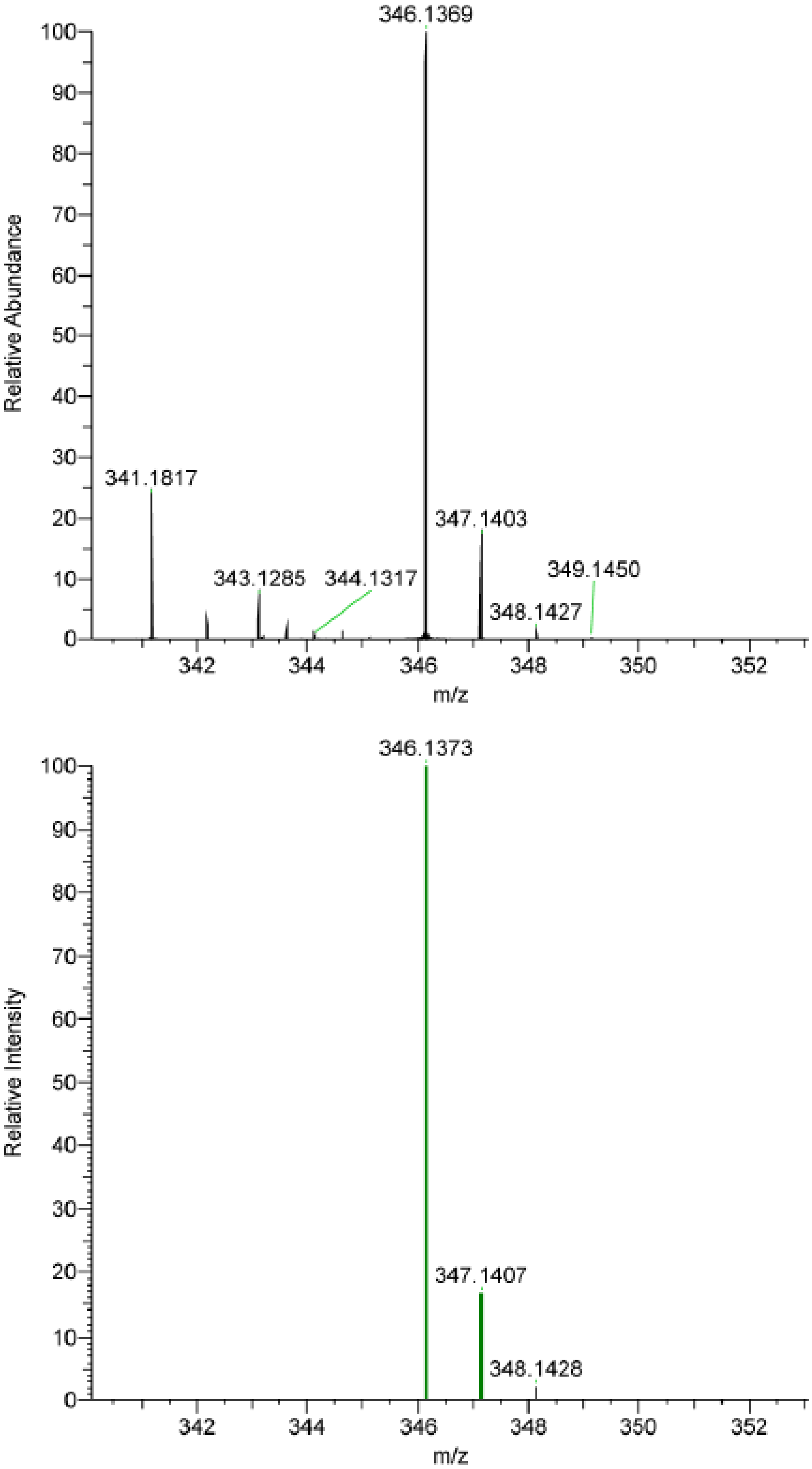

Figure S18 HR ESI-MS spectrum of 4’-Azidomethylbenzo-15-crown-5 (**5**).

1•XB

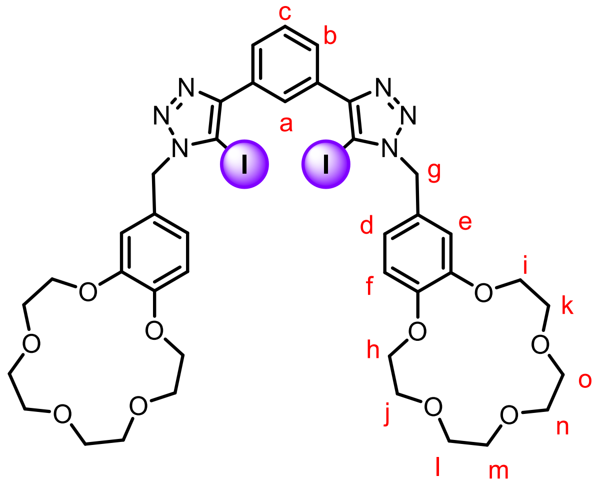

Purification Method: Wash with Et_2O (2x 10mL) and MeOH (2x 10mL).

^{1}H NMR (400 MHz, $CDCl_3$) δ = 8.52 (t, J = 1.8 Hz, $1H_a$), 7.98 (dd, J = 7.8, 1.8 Hz, $2H_b$), 7.55 (t, J = 7.8 Hz, $1H_c$), 6.93 – 6.79 (m, $6H_{d,e,f}$), 5.58 (s, $4H_g$), 4.16 – 4.05 (m, $8H_{h,i}$), 3.88 (m, $8H_{j,k}$), 3.74 (d, J = 2.8 Hz, $16H_{l,m,n,o}$).

^{13}C NMR (101 MHz, $CDCl_3$) δ = 150.02, 149.57, 149.48, 130.71, 129.05, 127.79, 127.27, 126.22, 121.31, 113.90, 113.70, 76.59, 71.24, 70.61, 69.65, 69.59, 69.17, 69.15, 54.41. (2 peaks missing due to coincident chemical shift).

HRMS (ESI +ve) m/z: 1025.1438, ([M+H]+, $C_{40}H_{47}I_2N_9O_{10}$ requires 1025.1438)

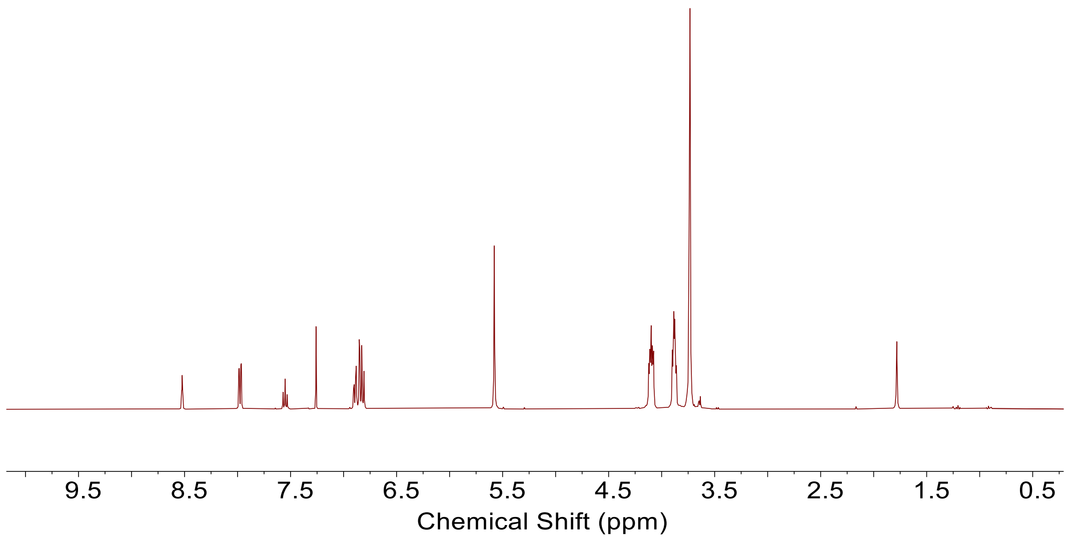

Figure S19 ^{1}H NMR spectrum of **1•XB**.

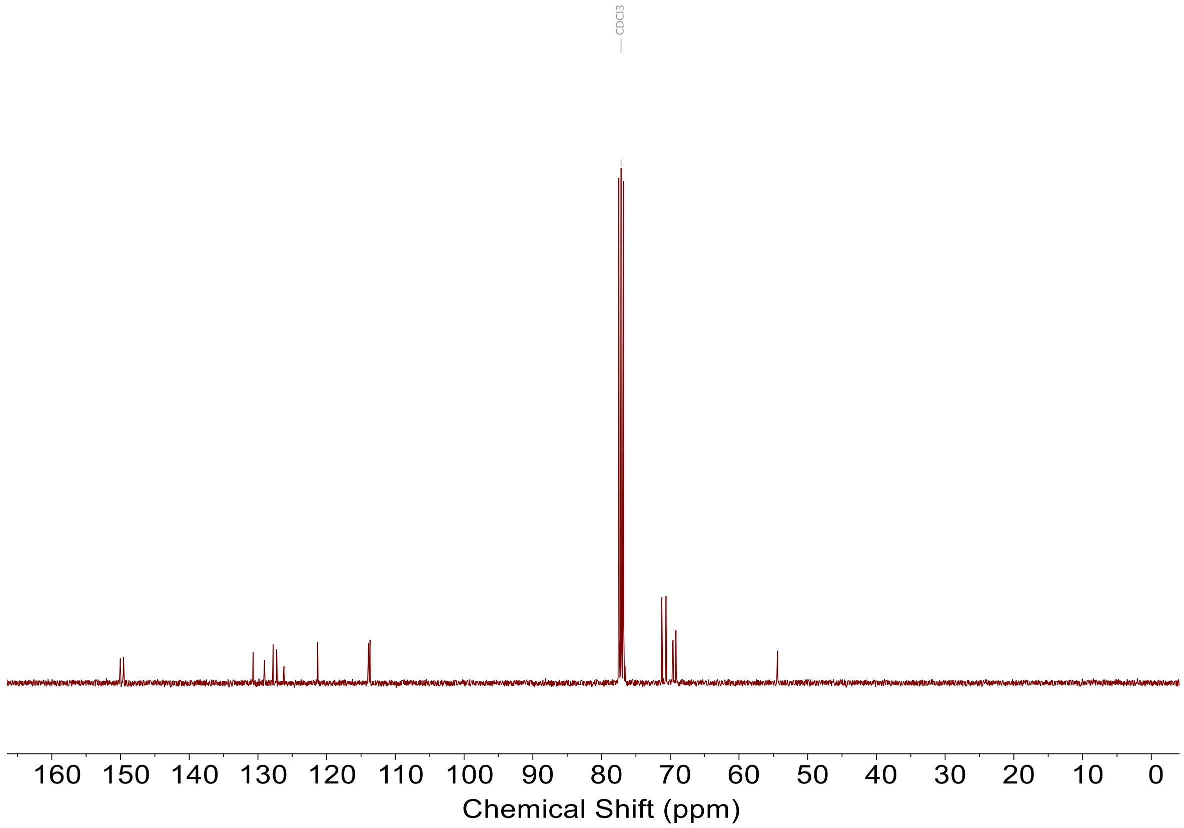

Figure S20 ^{13}C NMR spectrum of **1•XB**.

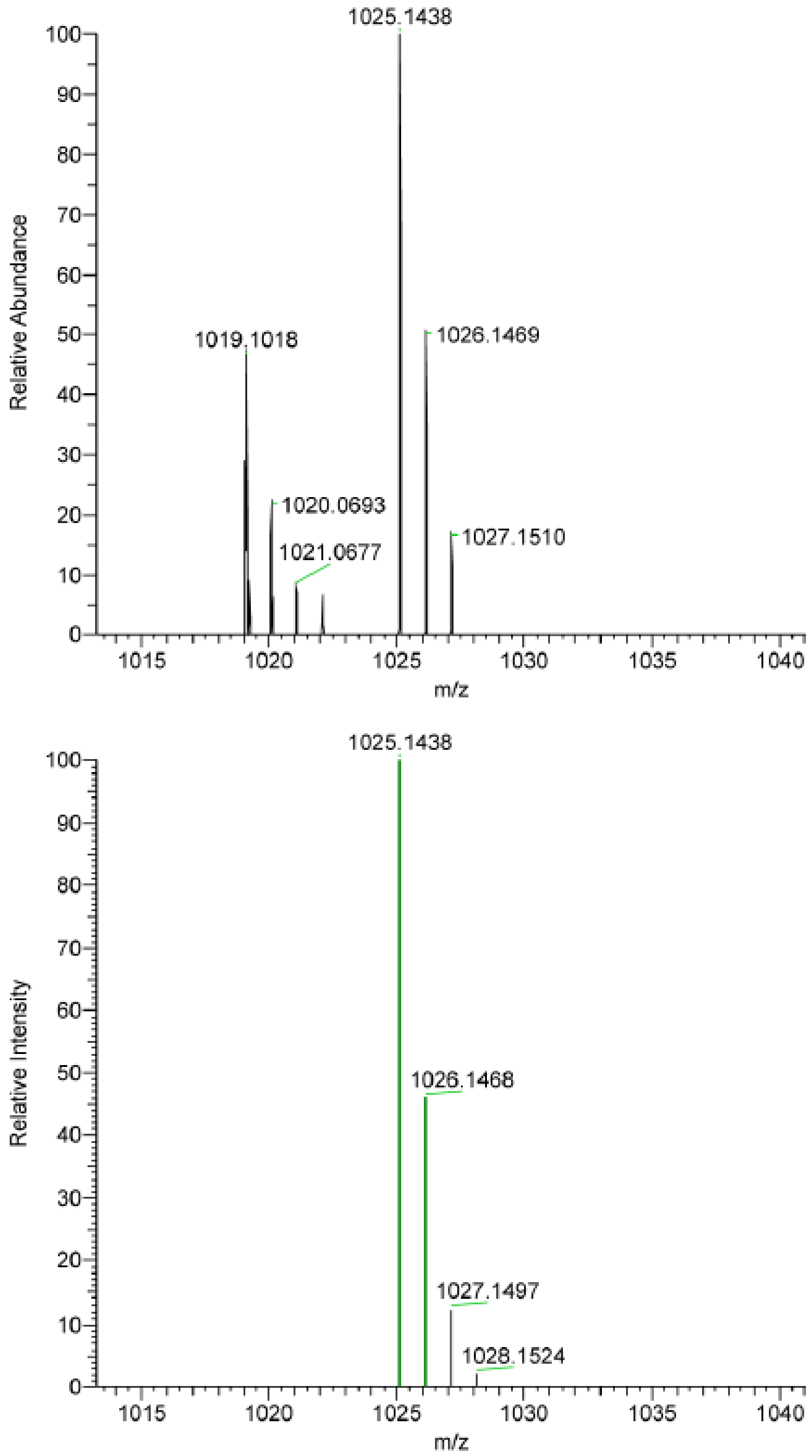

Figure S21 HR ESI-MS spectrum **1•XB**.

1•HB

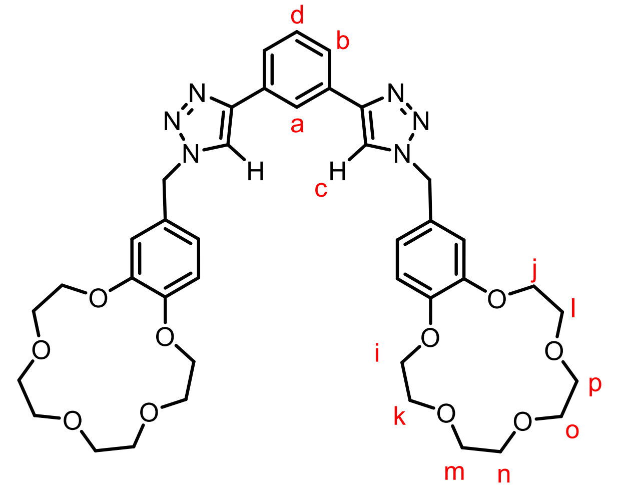

Purification Method: Wash with Et2O:MeOH 9:1 (*v/v*) (4x 10mL).

^{1}H NMR (400 MHz, $CDCl_3$) δ = 8.15 (t, J = 1.8 Hz, $1H_a$), 7.79 (dd, J = 7.7, 1.8 Hz, $2H_b$), 7.70 (s, $2H_c$), 7.45 (t, J = 7.7 Hz, $1H_d$), 6.93 – 6.79 (m, $6H_{e,f,g}$), 5.48 (s, $4H_h$), 4.19 – 4.06 (m, $8H_{i,j}$), 3.95 – 3.85 (m, $8H_{k,l}$), 3.80 – 3.73 (m, $16H_{m,n,o,p}$)

^{13}C NMR (126 MHz, $CDCl_3$) δ = 149.81, 147.91, 131.23, 129.53, 127.44, 125.49, 122.94, 121.54, 119.78, 114.14, 113.92, 71.24, 70.86, 70.60, 69.64, 69.57, 69.20, 69.18, 54.29.

HRMS (ESI +ve) m/z: 773.3497, ([M+H]+, $C_{40}H_{49}N_9O_{10}$ requires 773.3505)

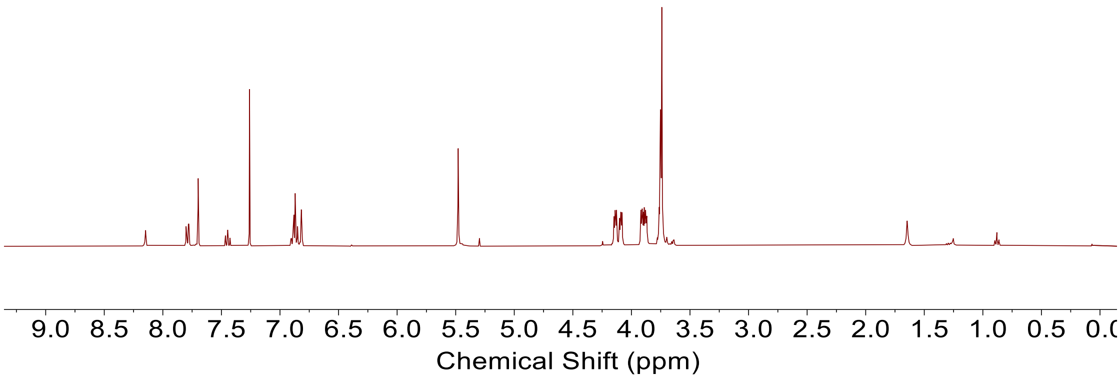

Figure S22 ^{1}H NMR spectrum of **1•HB**.

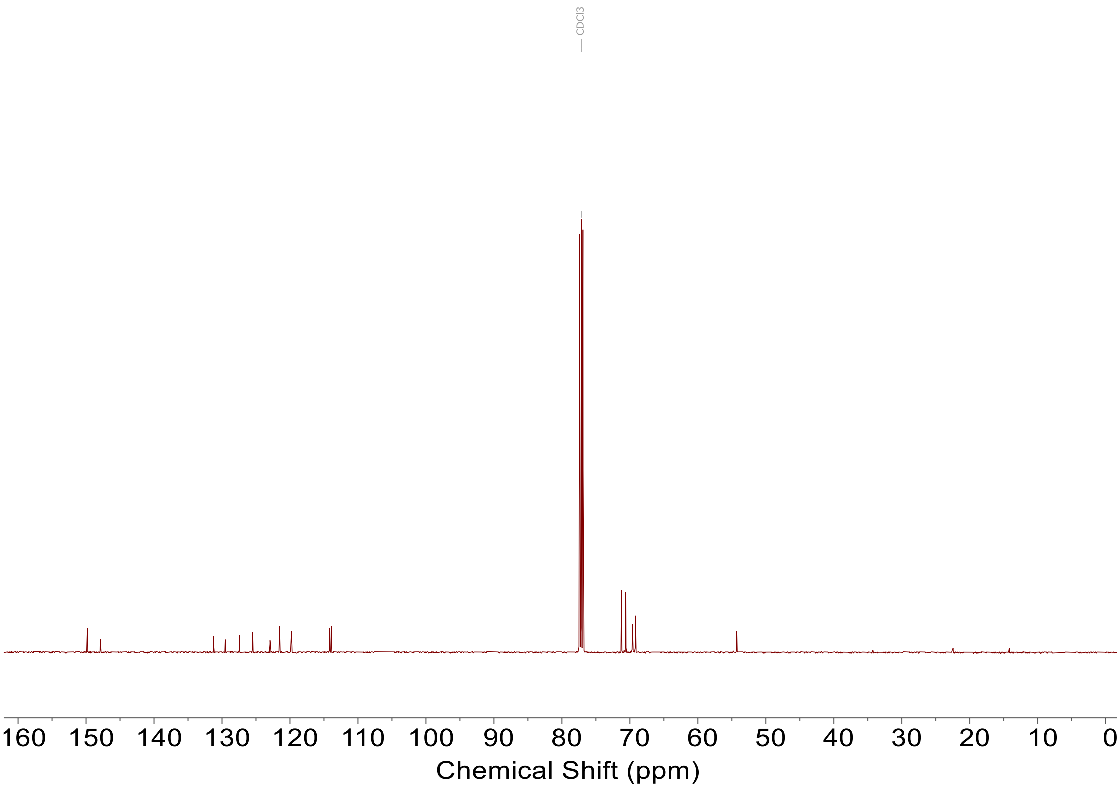

Figure S23 ^{13}C NMR spectrum of **1•HB**.

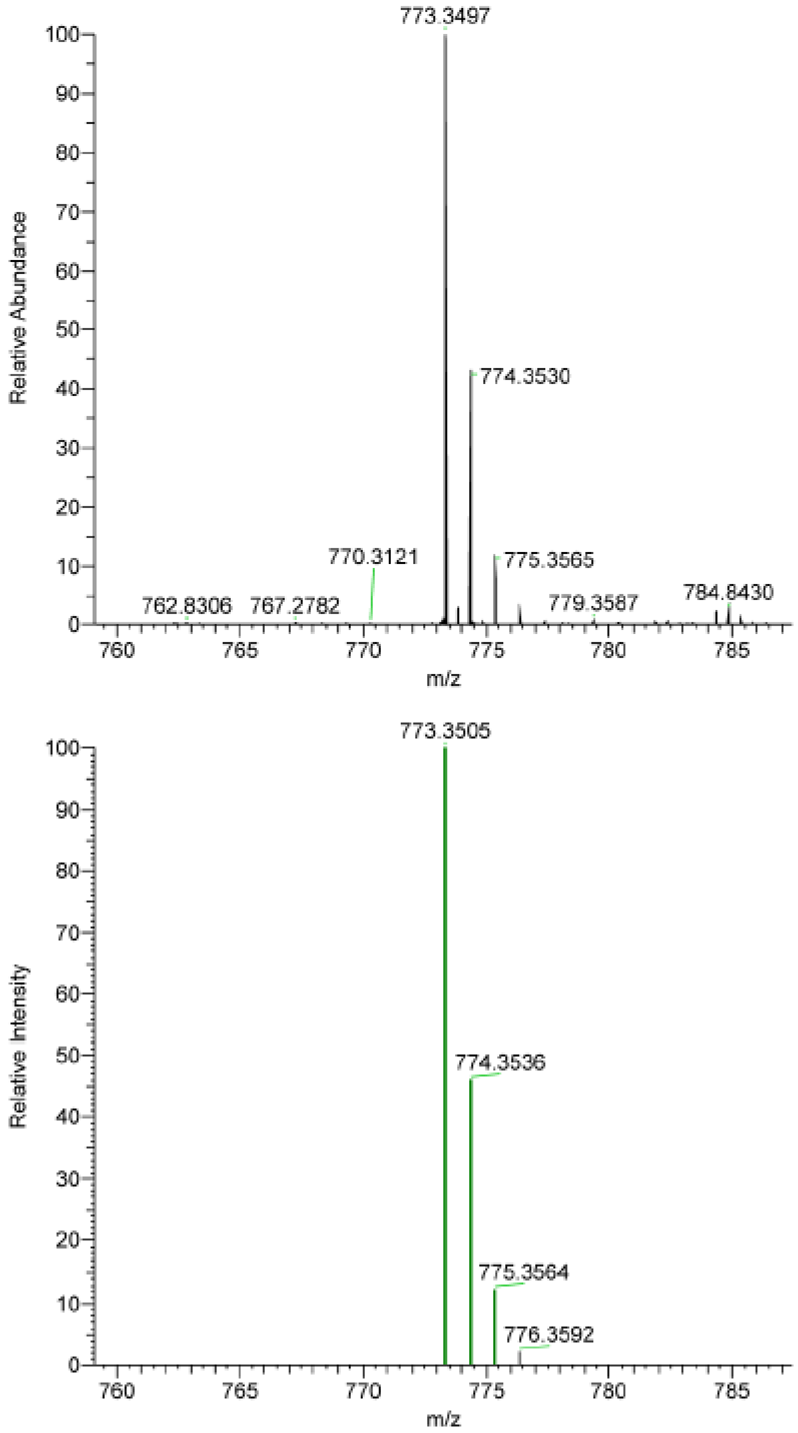

Figure S24 HR ESI-MS spectrum **1•HB**.
